# Functionalization of β-lactam antibiotic on lysozyme capped gold nanoclusters retrogress MRSA and its persisters following awakening

**DOI:** 10.1038/s41598-018-22736-5

**Published:** 2018-04-10

**Authors:** Sanjeeb Kalita, Raghuram Kandimalla, Ashim Chandra Bhowal, Jibon Kotoky, Sarathi Kundu

**Affiliations:** 1 Drug Discovery Lab, Institute of Advanced Study in Science and Technology, Paschim Boragaon, Assam Guwahati, 781035 India; 2 Soft Nano Laboratory, Institute of Advanced Study in Science and Technology, Paschim Boragaon, Assam Guwahati, 781035 India

## Abstract

In this study we have reported an efficient antibacterial hybrid fabricated through surface functionalization of lysozyme capped gold nanoclusters (AUNC-L) with β-lactam antibiotic ampicillin (AUNC-L-Amp). The prepared hybrid not only reverted the MRSA resistance towards ampicillin but also demonstrated enhanced antibacterial activity against non-resistant bacterial strains. Most importantly, upon awakening through cis-2-decenoic acid (cis-DA) exposure, the MRSA persister got inhibited by the AUNC-L-Amp treatment. Intraperitoneal administration of this hybrid eliminates the systemic MRSA infection in a murine animal model. Topical application of this nano conjugate eradicated MRSA infection from difficult to treat diabetic wound of rat and accelerated the healing process. Due to inherent bio-safe nature of gold, AUNC-L alone or in the construct (AUNC-L-Amp) demonstrated excellent biocompatibility and did not indicate any deleterious effects in *in vivo* settings. We postulate that AUNC-L-Amp overcomes the elevated levels of β-lactamase at the site of MRSA antibiotic interaction with subsequent multivalent binding to the bacterial surface and enhanced permeation. Coordinated action of AUNC-L-Amp components precludes MRSA to attain resistance against the hybrid. We proposed that the inhibitory effect of AUNC-L-Amp against MRSA and its persister form is due to increased Amp concentration at the site of action, multivalent presentation and enhanced permeation of Amp through lysozyme-mediated cell wall lysis.

## Introduction

Modern therapeutics are facing severe challenge against the rapid, ever-emerging frightening threat of multi-drug resistance (MDR) bacterial pathogens and failed to cope up with the morbidity and mortality associated with it^1^. According to World Health Organization (WHO), Methicillin-resistant *Staphylococcus aureus* (MRSA) infections increases the mortality rate of patients by 64% as compared to infections caused by the non-resistant forms^2^. The drug development pipeline has not kept pace with the ever increasing need of efficient novel antibiotics. Due to uncertainty in the path to market and profitability, majority of the pharmaceutical companies have abandoned the antibiotic development program^3^. Antibiotic tolerance phenomenon shown by the persister cell population adds further burden to the current antibiotic crisis. This subpopulation of phenotypically inactive variants of regular bacteria arises mainly as a response to stress through gene regulation cascades (toxin/antitoxin module systems)^4^.

The activity of traditional antibiotics (e.g., aminoglycosides, beta lactams, and fluoroquinolones) is restricted to only actively growing cells, whereas the metabolically inactive persister escapes the antibiotic action. Persister involvement in recalcitrance of chronic infections like tuberculosis and other biofilm associated infections were previously reported^5^. Superbugs that are resistant and tolerant to antibiotics are considered as a global epidemic and further deterioration will lead to the end of antibiotic era. Besides antimicrobial stewardship, novel therapeutic strategies and approaches are need of the hour to combat this consequential far-reaching problem^6,7^. In this regard, we are following an alternative, accelerated and cost effective approach to impair the bacterial resistance and restore the efficacy of old antibiotics. Though development of nanoantibiotics against microbial pathogens has been increasing since over the last decade, toxicity issues are restricting its utility for clinical applications^8^. Recent findings suggested that metallic nanoparticles in combination with antibiotics augments their antibacterial properties and thereby mitigate the cytotoxicity of both the agents by reducing the necessity for high dosages. Complex multifaceted antibacterial action mechanisms of metals reduce the probability of bacteria developing resistance towards the antibiotic conjugated metallic nanoparticle system^9,10^.

Gold nanoparticles are generally regarded as biologically inert and do not possess inherent antimicrobial property which can be exploited for augment the antibiotic efficacy^11–14^. Ampicillin (Amp) and other β-lactam antibiotics kill *Staphylococcus aureus* by hindering the function of cell wall penicillin-binding proteins (PBPs) 1 and 3. But this class of antibiotics is inefficacious against PBP2a, a transpeptidase enzyme used by Methicillin Resistant *S. aureus* (MRSA) to perform cell wall crosslinking functions^15,16^.

Combination of antibiotics with gold nanoparticles can restores its ability to destroy the bacteria that have acquired resistance towards them. The reason behind enhanced antibacterial activity was hypothesized as follows; (a) increase in the concentration of antibacterial at the site of antibiotic-bacterium interaction, (b) multivalent presentation of antibiotics, and (c) dysfunction of the bacterial efflux pump^17^. Gold nanoclusters (AUNCs) are attaining great attention in biomedical applications in last few years due to their unique characteristics including quantum mechanical behavior, excellent stability, high fluorescence intensity and good water solubility. Being ultra-small in size, AUNCs are explored as drug delivery vehicles that provide an interface for intense interactions with bacterial cellular and subcellular organelles^18^.

Method development for AUNCs synthesis has been explored with special attention towards exploitation of biological protein molecules as reducing and stabilizing agent in ‘one pot’ synthesis process of AUNCs^19^. The amino, carboxyl and thiol groups of proteins play the crucial role in the reduction and stabilization of AUNCs preventing the super atoms from aggregation^20^. Protein-mediated synthesis of AUNCs has been reported using Human Serum Albumin, Bovine Serum Albumin, transferrin, trypsin, apoferritin, horseradish peroxidase and lysozyme^21^. Lysozyme (1,4-β-N-acetylmuramidase) is a relatively small enzyme (14,307 Da mass) which is a part of the innate immune system and plays an important role in the prevention of bacterial infections by both muramidase dependent and independent (non-enzymatic membrane perturbing) mechanisms of action^22^. It is comprising of two domains and a cleft in the protein center which acts as the active site. The secondary structure of each domain differs significantly with one domain being mainly helical and the other is β-sheet in structure^21^.

Diabetes related chronic wounds including diabetic foot ulcers, pressure ulcers and venous leg ulcers are serious worldwide problem. Around 15% of diabetic patients develop lower extremity ulcers and 14–24% of them eventually undergo amputation^23^. Superficial infections and subsequent development of persistent biofilm of diabetic wounds (DW) hinder the normal phases of wound healing (hemostasis, inflammation, proliferation and remodeling). Due to patient compliance and recurrent use of antibiotics in the treatment of chronic diabetic wound infections leads to the development of bacterial resistance (MRSA, Vancomycin resistant *S. aureus*, etc.,) and further worsens the condition. Persister cell population of biofilm with antimicrobial tolerance obstructs the wound sterilization process and resulted in rapid regrowth of recalcitrant biofilms even after vigorous antibiotic treatment. Planktonic persister cells are reported to be less susceptible to antibiotics than static persister resides in biofilms. To regress the tolerant state of persister cells, several approaches have been attempted including the use of fatty acid signaling molecule cis-2-decenoic acid (cis-DA). In combination of antibiotics, it exhibited significant decrease in the persister cell population of *Escherichia coli* and *Pseudomonas aeruginosa* through escalation in their metabolic status without promoting their active growth^24^. In this present study, we evaluate the antibacterial effect of lysozyme capped gold nanoclusters (AUNC-L) functionalized with Amp (AUNC-L-Amp) on cis-DA pretreated MRSA persister. Further systemic MRSA infections and its associated complications resulted in high morbidity and mortality which demands prompted clinical attention. Community or hospital acquired multi-resistance MRSA (mrMRSA) infections can leads to irreversible damage. The prevalence of drug resistance strains is ever increasing, and hence treatment options (Vancomycin, Linezolid, etc.,) are getting limited to treat these chronic conditions.

The present study was aimed to investigate the antibacterial activity of AUNC-L-Amp against bacterial pathogens including MRSA clinical isolates in both metabolically active and inactive forms. We further confirm the activity of prepared nanohybrid against superficial and systemic MRSA infections in *in vivo* models. We hypothesized that functionalization of broad spectrum β-lactam antibiotic (Amp) with AUNC-L could restore its activity against MRSA and enhances the efficacy against non-resistant pathogens.

## Results

### Characterization of AUNC-L and AUNC-L-Amp

**Figure 1:**
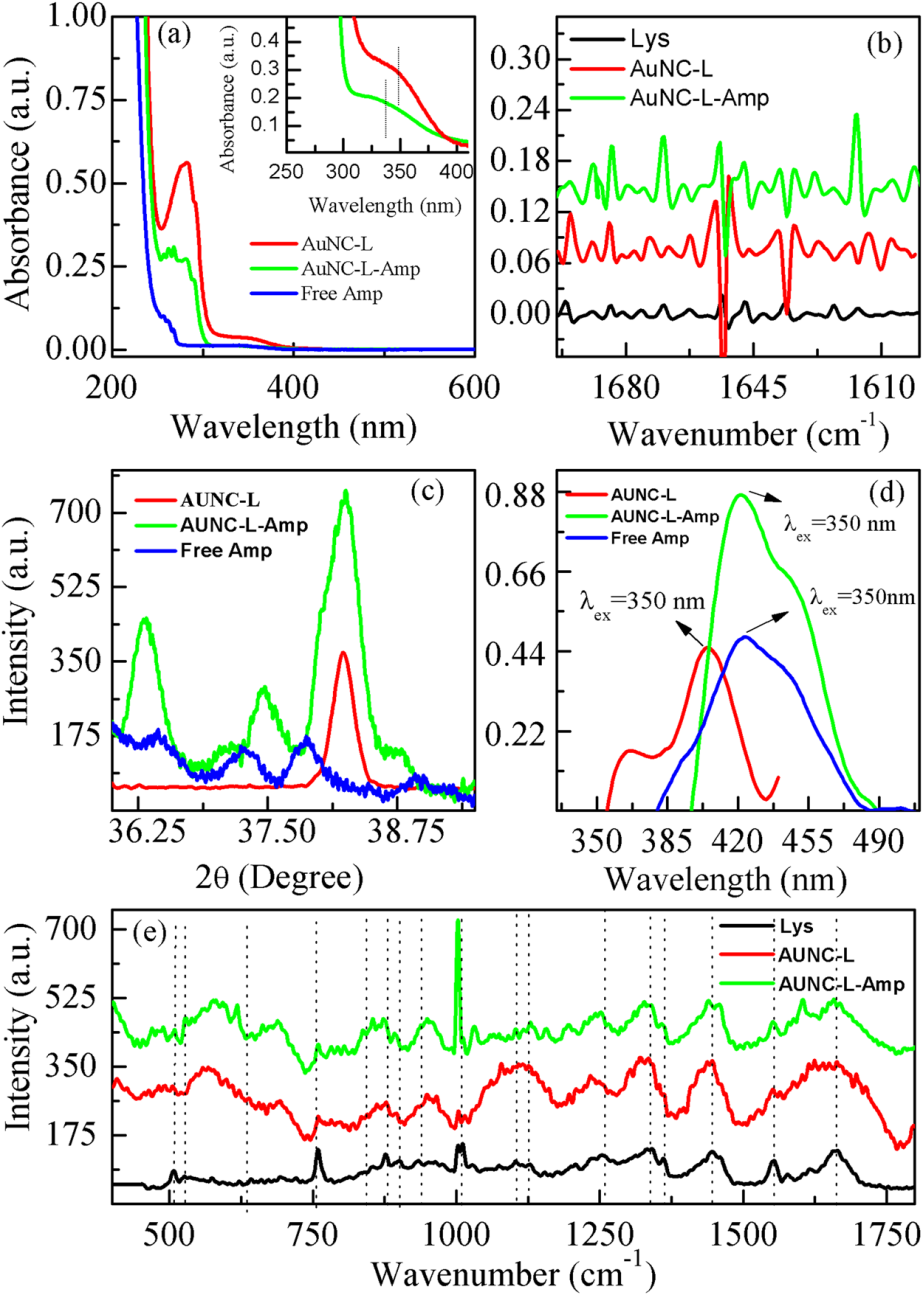
(**a**) UV-Vis spectra of AUNC-L, free ampicillin and ampicillin conjugated with AUNC-L (AUNC-L-Amp), inset is the zoomed image of AuNC-L and AuNC-L-Amp at 350 nm at elevated concentration. (**b**) FT-IR spectra of Lys, AUNC-L and AUNC-L-Amp showing second derivatives of amide-I bands (1700–1600 cm^-1^). (**c**) XRD spectra of AUNC-L, Free-Amp, and AUNC-L-Amp. (**d**) Fluorescence emission spectra of AUNC-L and AUNC-L-Amp. (**e**) SERS spectra of lysozyme, AUNC-L, and AUNC-L-Amp.

In this study, we have synthesized lysozyme capped gold nanoclusters (AUNC-L) which were confirmed by the presence of characteristic absorption peak of gold nanoclusters at 350 nm as shown in Fig. 1(a) ^9,25^. The absorption peak of free ampicillin (Amp) was observed at 264 nm as shown in Fig. 1a. The absorption peaks obtained from AUNC-L-Amp, i.e., after the conjugation between free Amp and AUNC-L observed at 343 nm (inset Fig. 1a). The peak at 278 nm is due to presence of lysozyme protein and the observed oscillation near the absorption peak of protein (green curve) confirms the successful conjugation of ampicillin on AUNC-L surfaces. The inset in Fig. 1a shows the zoomed images of AUNC-L and AUNC-L-Amp absorption peaks at elevated concentrations.

**Figure 2:**
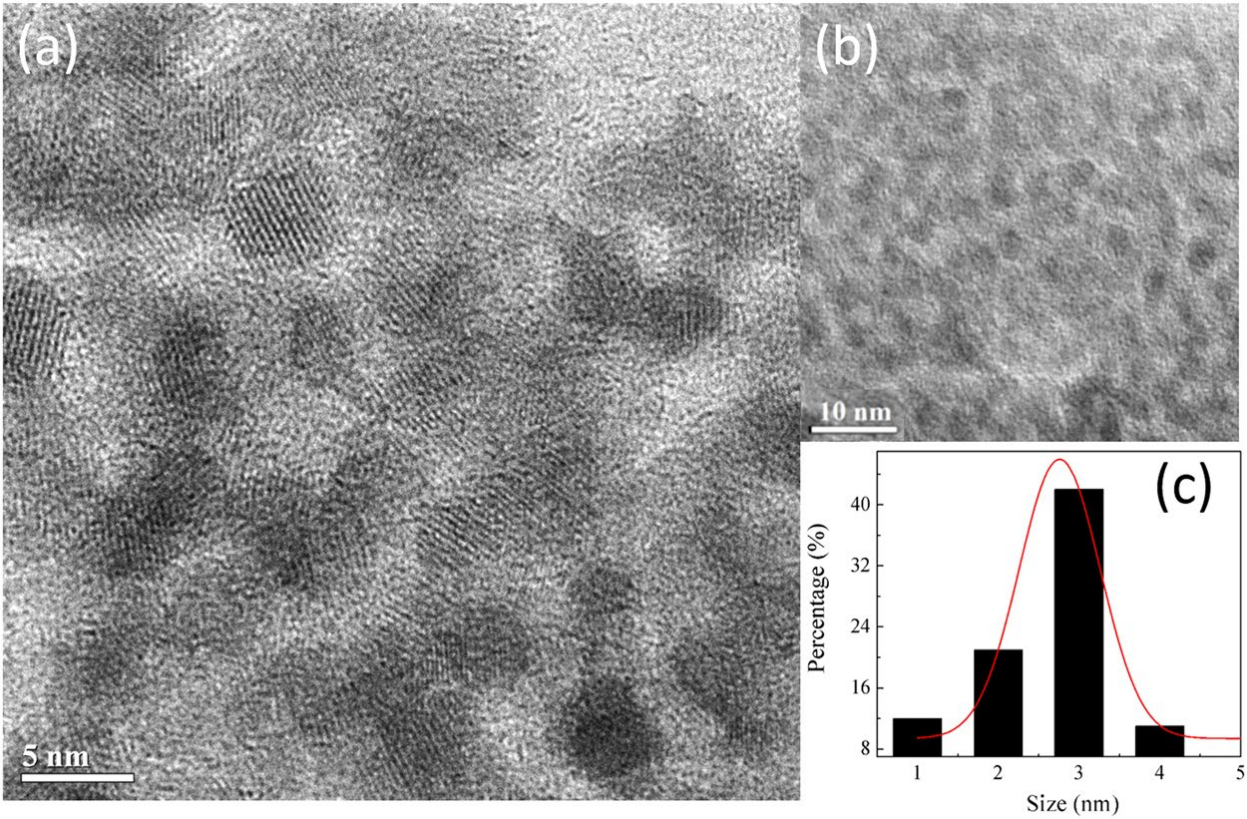
(**a**) HR-TEM image showing crystal lattice of AUNC-L-Amp; (**b**) TEM image of AUNC-L-Amp; (**c**) Particle size distribution of AUNC-L-Amp.

Transmission electron microscopy (TEM) analysis provides details about size and morphology of AUNC-L-Amp (Fig. 2). Solid, spherical and ultra-small sized nanoclusters with the diameter ranging from (1–5 nm) were recorded for AUNC-L-Amp (Fig. 2b). From the size distribution histogram (Fig. 2c), the average size of AUNC-L-Amp was calculated as 2.71 ± 0.15 nm. High-resolution TEM (HRTEM) suggests the crystalline nature of AUNC-L-Amp.

Dynamic light scattering (DLS) measurements revealed the hydrodynamic sizes of AUNC-L and AUNC-L-Amp as ≈6.5 and 11 nm respectively which supports the successful conjugation. The change in zeta potential 33.6 mV (AUNC-L) to 26.7 mV (AUNC-L-Amp) also indicates the Amp loading on AUNC-L^26^. The steady state fluorescence study reveals that AUNC-L displays fluorescence emission at ≈407 nm against excited wavelength of 350 nm^25^. Whereas, for the conjugated system (AUNC-L-Amp) the fluorescence emission was observed at ≈420 nm against excitation wavelength of 350 nm Fig. 1d.

Infrared spectroscopy (IR) is one of the most convenient and efficient methods for characterizing the lysozyme conformational changes^27^. Lysozyme’s amide-I (1700–1600 cm^−1^) and amide-II (1480–1575 cm^−1^) bands are the most prominent vibrational bands of its backbone. Amide-I region is most sensitive region and changes in conformation leads to changes in its secondary structures which is visible within the IR range of 1700–1600 cm^−1^. In this study, a total of 16 peaks were assigned for both AUNC-L and AUNC-L-Amp (Fig. 1b, Fig. S1 & Table. S1). Peaks were assigned based on the beta sheet, beta turn, anti-parallel beta sheet, intra and inter- molecular beta strand, alpha helix, and random coil conformation^28,29^.

XRD spectrum Fig. 1c from AUNC-L shows a peak at 2θ ≈ 38.25° which corresponds to (111) crystal plane of fcc gold nanostructures. Diffraction peaks of Amp imply its poly crystalline nature^30,31^. AUNC-L-Amp showed the characteristic peak of gold nanostructures (2θ≈38.25°) and Amp whereas the peak positions of Amp were modified which confirms the conjugation^17^. Surface-enhanced Raman spectroscopy (SERS) detects vibrational changes take place into the molecular level and determines the conformational changes occur in protein secondary structures^32^. The band at 930 cm^−1^ regions was assigned as C-C stretching of α-helix structure of lysozyme which was shifted to 935 cm^−1^ after nanoclusters formation and further shifted to 937 cm^−1^ on conjugation with Amp. The amide-I band of lysozyme at 1660 cm^−1^ was shifted to 1662 cm^−1^ in both AUNC-L and AUNC-L-Amp. Further, a red-shift of amide-III band of lysozyme was observed from 1255 cm^−1^ to 1250 cm^−1^ in both AUNC-L and AUNC-L-Amp. The S-S stretching peak of lysozyme at 507 cm^−1^ was shifted to 509 cm^−1^ in both AUNC-L and AUNC-L-Amp due to dissociation of disulfide bonds^33^. Characteristic vibrational bands of Amp (1002, 1330, 1448, 1494 and 1604 cm^−1^) were also observed in Raman spectra of AUNC-L-Amp which confirms the successful conjugation of Amp on AUNC-L (Fig. 1e)^34^.

### Biocompatibility of AUNC-L and AUNC-L-Amp

#### Hemocompatibility

**Figure 3:**
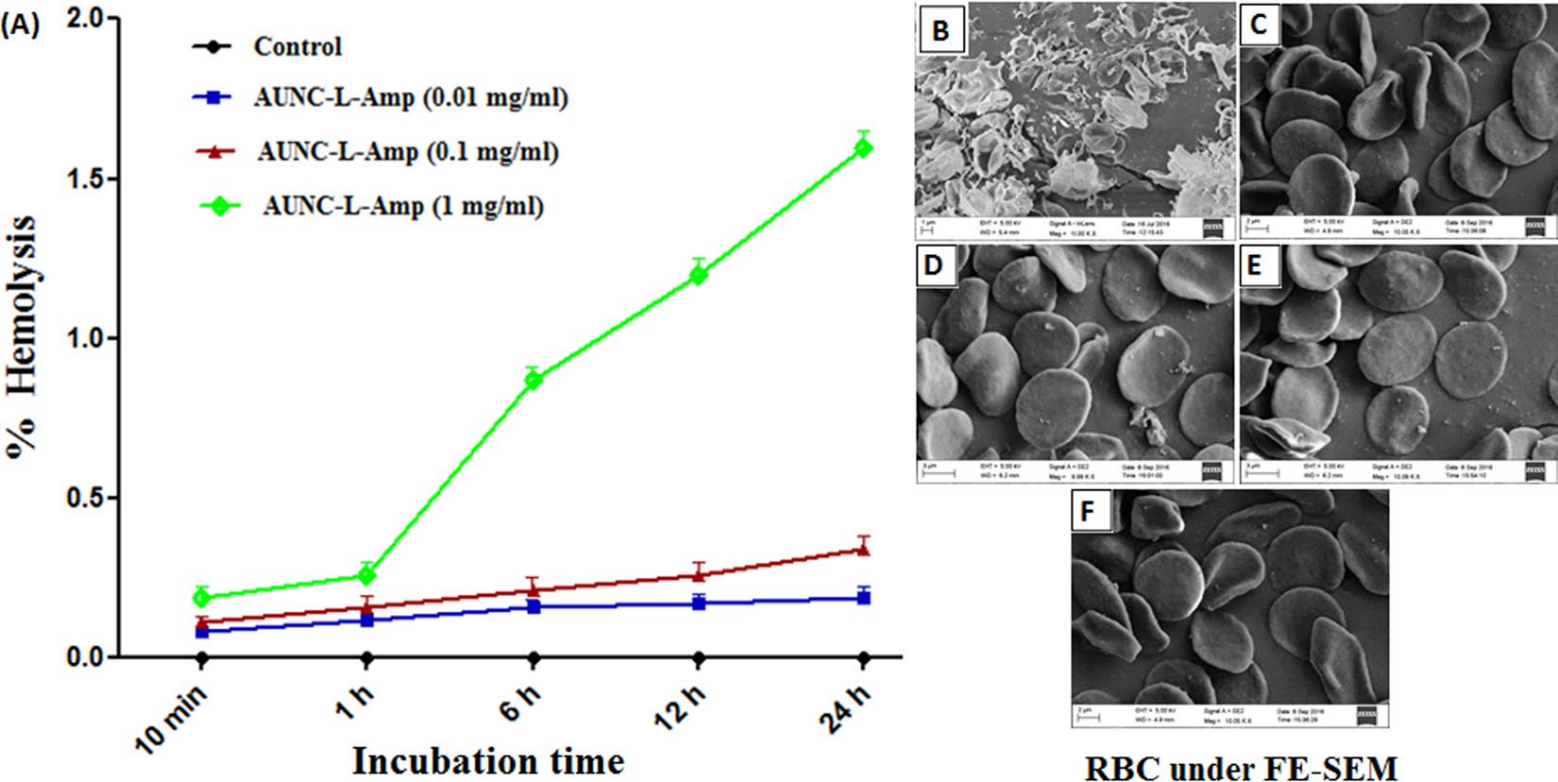
(**A**) Effect of various concentration of AUNC-L-Amp on % hemolysis of human RBC at various time points; Morphology of RBC treated with distilled water (**B**), PBS (**C**) and AUNC-L-Amp (0.01, 0.1 & 1 mg/ml respectively) (**D**–**F**) under FE-SEM.

Hemolytic potential has been considered as a crucial parameter for biocompatibility evaluation of various blood-contacting biomedical materials. Hemolysis refers to the disturbance of RBC membrane integrity which results in hemoglobin release from RBC. In this present study, RBCs were exposed to different concentrations of the AUNC-L-Amp (0.01, 0.1 & 1 mg/ml) for 10 min, 1 h, 6 h, 12 h and 24 h (Fig. 3A). AUNC-L-Amp at 0.01 & 0.1 mg/ml concentrations exhibited negligible hemolysis even after 24 h of incubation. However, at the tested highest concentration (1 mg/ml), the RBCs started to lyse from 1 h, reaching 1.6% hemolysis at 24 h which is in the permeable limit (5%)^35^. It was observed that RBC lysis in the presence of AUNC-L-Amp was dose and time dependent. The results confirmed that AUNC-L-Amp does not disturb the RBCs membrane integrity substantially. FE-SEM analysis of the RBCs revealed that even after 24 h of incubation, AUNC-L-Amp at all the concentrations tested did not alter the RBC morphology and maintained its natural shape of biconcave discs (Fig. 3B–F). Whereas the RBCs treated with distilled water were completely lysed or distorted.

#### Cytocompatibility

To evaluate the cytocompatibility, MTT assay was performed. After 24 h of incubation with AUNC-L-Amp (up to 250 µg/ml) L929 cells exhibited a considerably higher viability (80.7%) compared to Free-Amp (78.4%) whereas bare AUNC-L exhibited the highest viability (86.2%) towards the tested cell lines (Fig. S2). A majority of gold (I) and gold (III) compounds reported to exhibit varying degrees of toxicity towards mammalian cells^36^. In this present study, we found that AUNC-L-Amp was biocompatible towards mammalian cells and it reduces the toxicity of the drug candidates upon conjugation. Before proceeding to the pre-clinical evaluation of AUNC-L-Amp, high dose tolerance demonstrated by mammalian cell line is a prerequisite. Colony forming assay results revealed that treatment with bare AUNC-L, AUNC-L-Amp and Free- Amp (250 µg/ml) on L929 cell line for 24 h did not induce considerable cytotoxicity as evident from the survival fraction values of 95.3, 93.7 and 92.4 respectively for the tested samples.

### Isolation and confirmation of persister cell state

To generate MRSA persister, MRSA1 and MRSA2 were cultured up to stationary phase and treated with 20 μg/mL (10× MIC) gentamicin for 4 h^37^. Even after the treatment, the concentration (~10^10^ CFU/ml) of MRSA population in stationary phase was not decreased (Fig. S3). The gentamicin-tolerant cells were further treated for additional 4 h with 10× MIC of gentamicin, vancomycin and ciprofloxacin to determine the tolerance of MRSA1 and MRSA2 towards other antibiotics. After 4 h of treatment, none of the antibiotic decreases the viability of the gentamicin-tolerant cells (Fig. S3). These results suggested that all of the stationary phase population of MRSA1 and MRSA2 are in a persistent state, which is in agreement with previous reports^37,38^.

### *In vitro* anti-bacterial activity of Free-Amp, AUNC-L and AUNC-L-Amp

**Figure 4:**
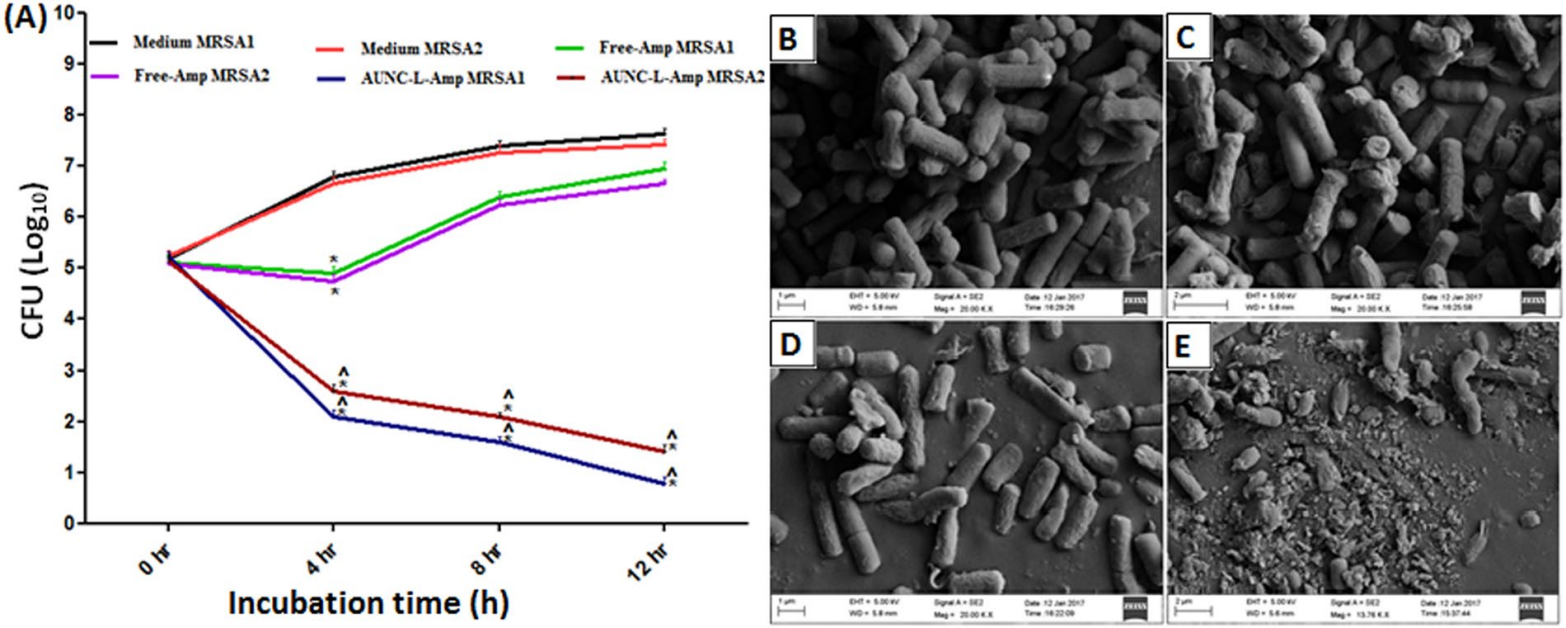
(**A**) *In vitro* killing kinetics of MRSA1 & 2 on treatment with Free-Amp and AUNC-L-Amp. All the results were expressed in mean ± S.D. *p ≤ 0.05 in comparison with medium MRSA1 & 2. ^p ≤ 0.05 in comparison with Free-Amp MRSA1 & 2 respectively; Surface morphology of MRSA under FE-SEM on treatment with PBS (**B**), AUNC-L (**C**), Free-Amp (**D**) and AUNC-L-Amp (**E**).

Antibacterial assay results demonstrated a significant enhancement (50–89% fold increase) in antibacterial activity of AUNC-L-Amp compared to Free-Amp in terms of zone of inhibition against 9 nonresistant bacterial pathogens (Table. S2). Fascinatingly, Amp loading on AUNC-L enables its activity against 10 MRSA clinical isolates, where Free-Amp and AUNC-L did not exhibit considerable activity (Table. S3). MIC/MBC values exhibited by AUNC-L-Amp against the tested nonresistant bacterial pathogens were significantly lower than that of Free-Amp (Table. S4), whereas Free-Amp functionalization on AUNC-L (AUNC-L-Amp) retrieves the antibacterial activity of Amp against 10 MRSA clinical isolates (Table. S5). MRSA growth was not inhibited up to 5 mg/ml concentration of Free-Amp. For anti-MRSA activity studies, vancomycin was kept as standard which shows variable zone of inhibition (33–51 mm) and MIC/MBC values (0.5–2 µg/ml) against the tested MRSA strains. The AUNC-L-Amp retarded the biofilm formation by 95%, 95%, 92% & 88% respectively for the tested MRSA1, MRSA2, MRSA3 & MRSA4 strains where, there is no considerable inhibition of biofilm formation was observed in the case of Free-Amp (Table. S6). Since MRSA1 & 2 were found to be more susceptible to AUNC-L-Amp so these two strains were continued for further evaluations. AUNC-L-Amp, exhibited intensified killing rate against MRSA1 & 2at different time points (4^th^, 8^th^ & 12^th^ hour) whereas, Free-Amp obstructed the bacterial growth up to the 4^th^ hour (Fig. 4A). *In vitro* assay results were also supported by FE-SEM analysis of MRSA1; where treatment with AUNC-L and Free-Amp did not exhibit an alteration in the bacterial morphology. On contrary to this, AUNC-L-Amp treatment destructed MRSA cellular structures (Fig. 4B–E). Confocal laser scanning microscopy analysis (CLSM) revealed the efficient penetration of AUNC-L-Amp and AUNC-L into the MRSA cytosol. Treatment of AUNC-L failed to kill the MRSA, whereas significant death was recorded in AUNC-L-Amp treated group (Fig. S4). Table. S7 summarizes the MICs at the 1^st^, 5^th^, 10^th^ & 15^th^ exposure of sub-lethal dose of both Free-Amp and AUNC-L-Amp against tested bacterial strains. The results of the study revealed that repeated exposure of Free-Amp resulted in four-fold increase of MIC value whereas in the case of AUNC-L-Amp, MIC value remains constant. Most importantly the tested MRSA strains failed to attain resistance against AUNC-L-Amp even after 15^th^ exposure. Zone of inhibition, MIC, MBC, killing kinetics and biofilm inhibition assay results confirmed the regression of MRSA resistance and greater anti-bacterial activity of AUNC-L-Amp against nonresistance strains (Gram + Ve & − Ve).

### SYTOX Green membrane permeability assay for MRSA persister

**Figure 5:**
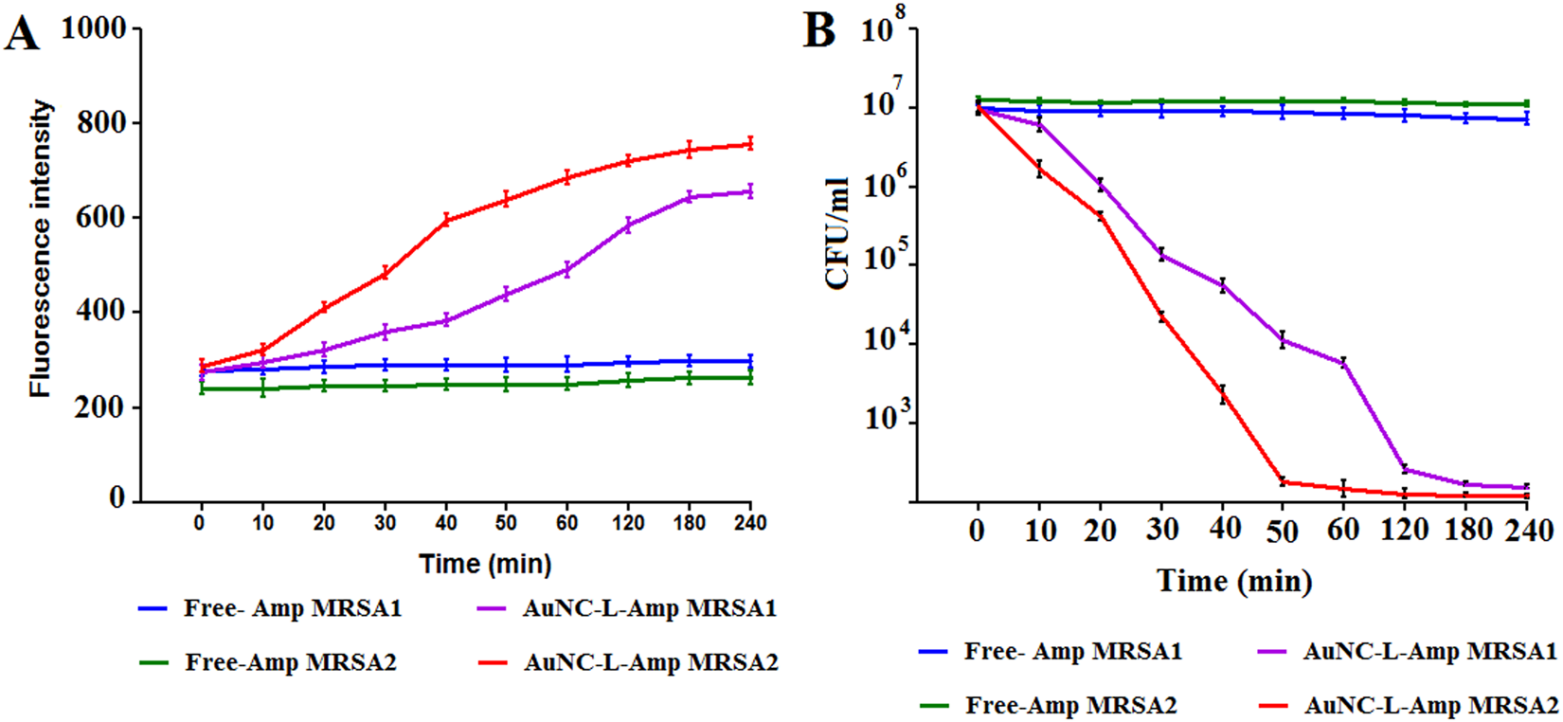
AUNC-L-Amp kill MRSA persisters by inducing membrane permeabilization. MRSA1 and MRSA2 persisters were treated with Free Amp and AUNC-L-Amp separately. (**A**) Membrane permeabilization was measured spectrophotometrically by monitoring the uptake of SYTOX Green (excitation wavelength of 485 nm and an emission wavelength of 525 nm). (**B**) Colony forming unit counts of persisters were measured by serial dilution and plating on nutrient agar plates. Results are shown as means ± s.d. n = 3.

Membrane permeabilization through direct or indirect means of cell damage is the main mode of action for several potent antibiotics such as HT61, lysostaphin and nisin^39^. As intact cell envelope is crucial for vitality of both actively dividing cells and persister, we assumed that cell wall penetrating nanopaticulate system can be an effective means for drug delivery against MRSA persister. In order to evaluate AUNC-L-Amp’s potential in membrane permeabilization mediated killing of MRSA persister, reporter dye (SYTOX green) based assay was performed. Bacterial cells with permeabilized membranes can uptake the SYTOX Green and binding of same to DNA shows >500-fold signal enhancement^40^. As expected, AUNC-L-Amp induced substantial membrane permeabilization as measured up to 4 h (at definite time intervals), whereas the Free-Amp failed to do so in tested MRSA persister (Fig. 5A).

### Effect of AUNC-L-Amp on Cis-DA exposed MRSA persister

Recent reports suggested that cis-DA exposure mediated awakening of persister of *Pseudomonas aeruginosa* and *Escherichia coli* increases their susceptibility towards the co-administered antibiotics with a substantial decrease in viability to the point of eradication^24^. In this study, we evaluated free Amp and AUNC-L-Amp’s ability to kill MRSA persister in presence of cis-DA (310 nM) for a period of 4 h. A significant (P < 0.001) decline in persister cell viability was observed for AUNC-L-Amp as compared to Free Amp treatment (Fig. 5B). These findings demonstrate that Cis-DA alters the metabolic status of MRSA and increase its susceptibility towards AUNC-L-Amp.

### Effect of AUNC-L-Amp on systemic MRSA infection of mice

**Figure 6:**
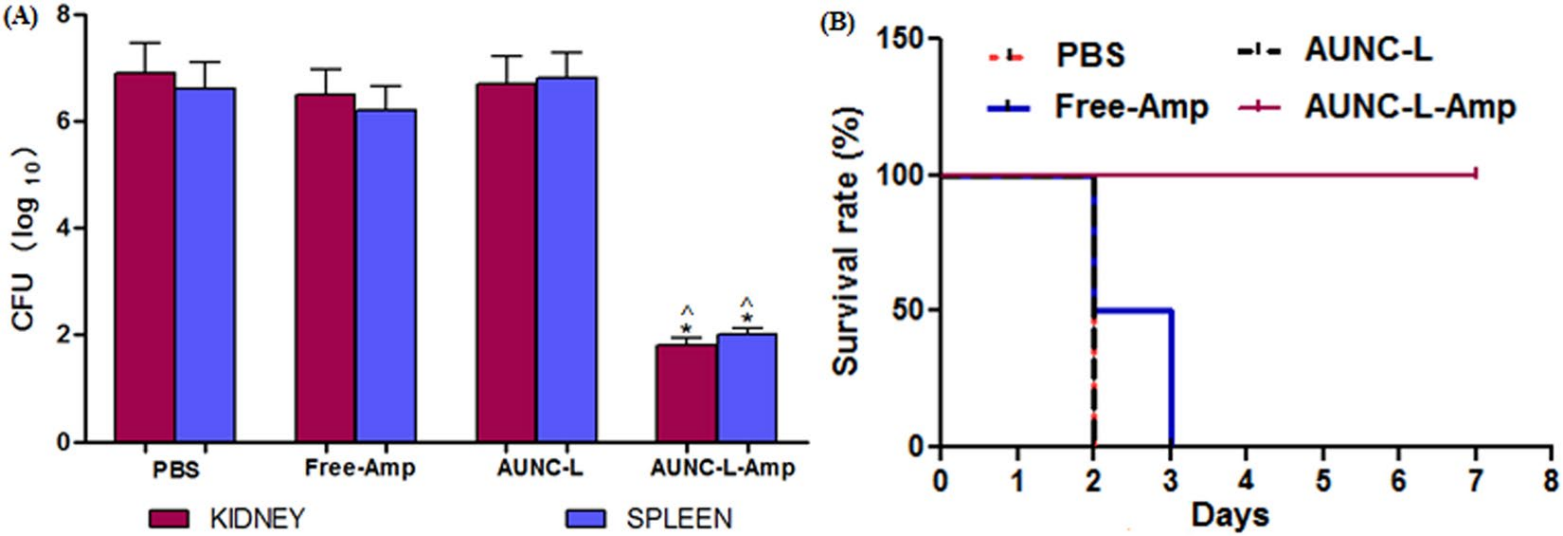
(**A**) MRSA load by means of CFU on kidney and spleen post 23 h of drug treatment. All the results were expressed in mean ± S.D. *p ≤ 0.05 in comparison with PBS treated group. ^p ≤ 0.05 in comparison with Free-Amp treated group; (**B**) Kaplan-Meier curve showing percentage survival rate of MRSA-infected mice on various drug treatments.

From *in vitro* killing kinetics assay, MRSA1 was found to be most susceptible towards AUNC-L-Amp and hence selected for this study. At 24 h post infection (23 h of post-treatment) the CFU burden in the kidney and spleen of the mice were estimated. AUNC-L-Amp demonstrated significant (p < 0.05) reduction (4.5–5.1 logs) of MRSA burden in both kidney and spleen compared to PBS treatment. Free-Amp and AUNC-L treatment did not reduce the MRSA burden (Fig. 6A). The AUNC-L-Amp system demonstrated clearance of MRSA from vital organs like kidney and spleen, which resulted in increased survival rate. Further, the survival rate at day 7 post-MRSA infection was significantly higher in the AUNC-L-Amp treated group compared with the Free-Amp, AUNC-L and PBS treated groups (Fig. 6B).

### Effect of topical application of AUNC-L-Amp on wound healing in MRSA infected diabetic wounds

**Figure 7:**
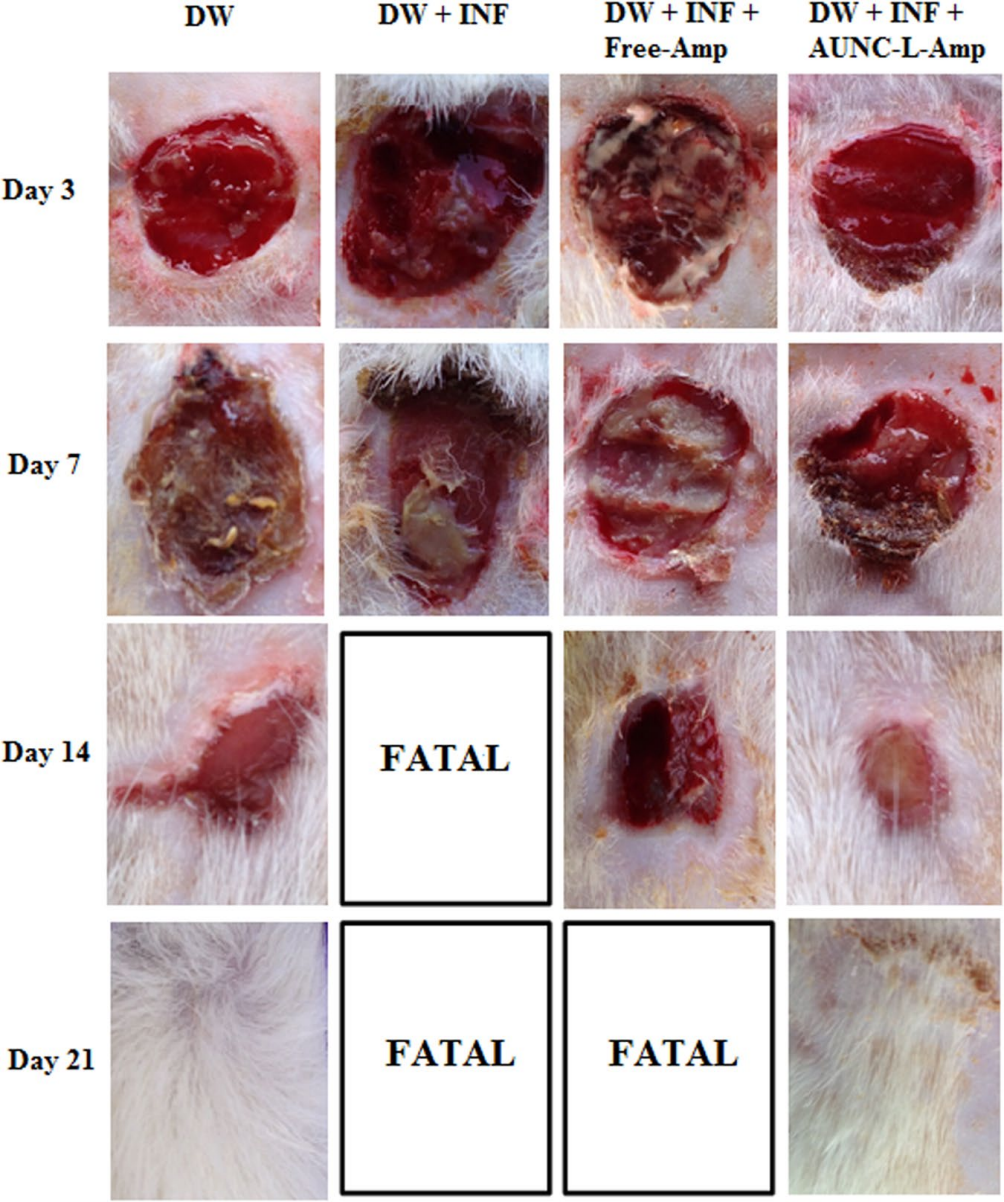
Wound healing progression of diabetic wounds over time. Abbreviations: DW: Diabetic wound; INF: MRSA infection.

**Figure 8:**
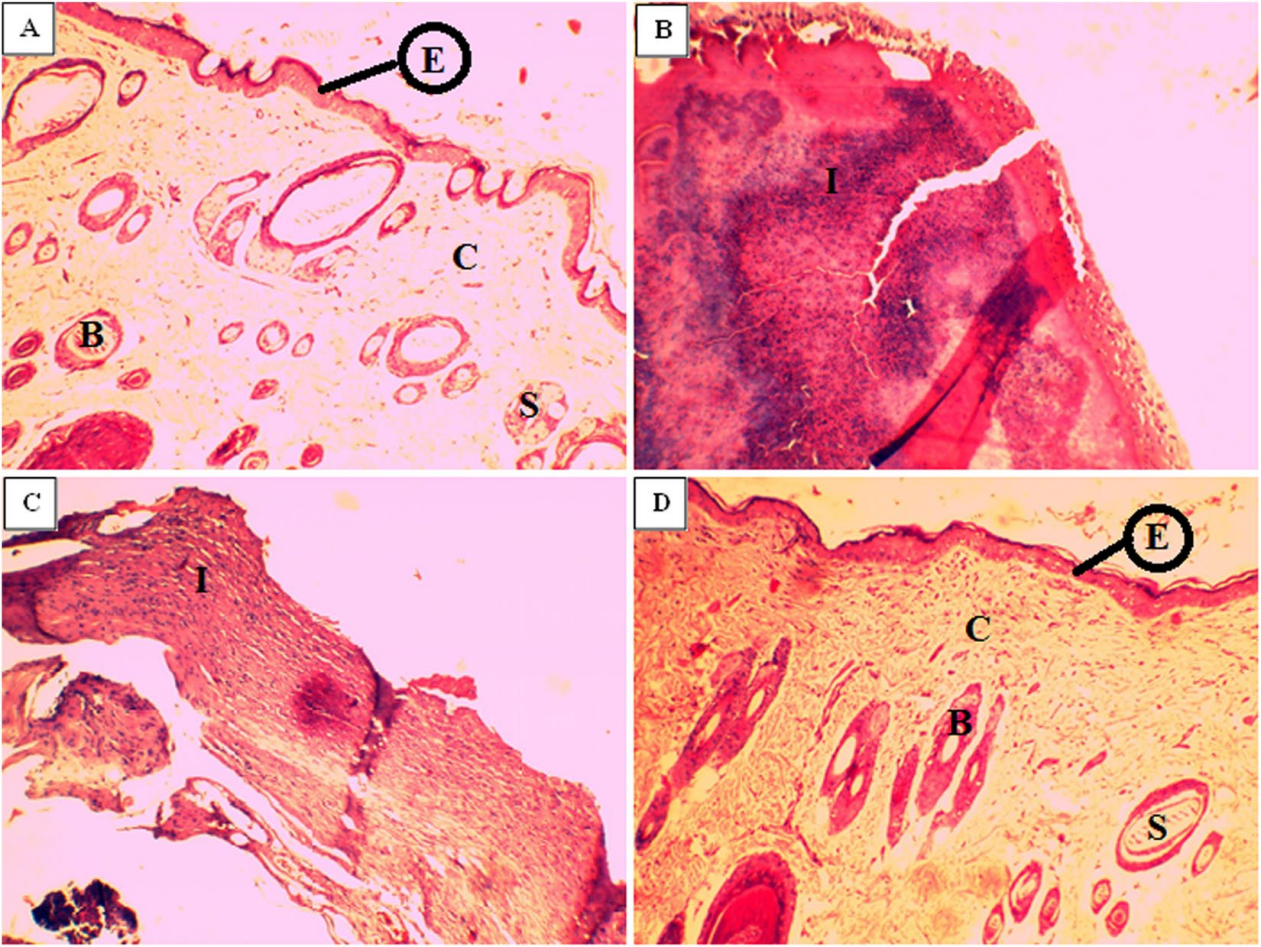
Histopathology of wounded tissue from different treatment groups; (**A**) Normal DW (**B**) DW with MRSA infection (**C**) MRSA infected DW treated with Free-Amp (**D**) MRSA infected DW treated with AUNC-L-Amp. Abbreviations: **DW:** Diabetic wounds; **E:** Epidermis; **C:** Collagen tissue; **B:** Blood vessels; **S:** Sweat gland; **I:** Inflammatory cells.

Significant (p ≤ 0.05) reduction in MRSA load in terms of CFU count (2.6 to 7.5 logs) at different time intervals (3^rd^, 7^th^, 14^th^ & 21^st^ day) was observed in AUNC-L-Amp treated MRSA infected DW compared to PBS treatment. Free-Amp treatment failed to reduce the MRSA load on DW (Fig. S5). Pronounced wound healing was observed in AUNC-L-Amp treated animals which were comparable to non-infected DW. Mortality was recorded in PBS & Free-Amp treated animals during 10 to 17 days of post-infection (Fig. 7). Further, AUNC-L-Amp treatment normalized the abnormal inflammatory cytokines (IL-1β & TNF-α) whereas no difference was observed in other treatment groups (Figs S6 and S7). Histopathology results of wound area on 21^st^ day further strengthen the claim as the dermal ultra-structure was better organized with prominent epithelialization and distinct acanthotic maturing sub-epidermis in AUNC-L-Amp treated animals. In the case of PBS (on the 13^th^ day) and Free-Amp (on the 17^th^ day) treated animals showed complete disruptions along with inflammatory cell infiltrate (Fig. 8).

## Discussion and Conclusion

Conventional antibiotics with typical mechanism of actions are gradually getting resisted by MRSA at a faster pace. In this regard, researchers are pursuing development of innovative facile strategies to formulate accelerated, cost effective, alternative approaches for the effective treatment of resistant bacterial infections. The dearth in treatment options encourages the efficacy restoration or enhancement of inefficacious or limited efficacious antibiotics through integrating the same with nanoscalic entities. In this regard the present study demonstrates the functionalization of widely used β-lactam antibiotic Amp (as a model drug) on AUNC-L to combat MRSA and other non-resistant bacterial pathogens.

Lysozyme is an antibacterial enzyme which acting as reducing and capping agent in the synthesis of AUNC-L. Further Amp molecule was subsequently surface functionalized on AUNC-L; predominantly through thioether moiety mediated partial covalent interaction (Au-S-Amp and Au-N-Amp bonds) and electrostatic interaction between negatively charged Amp and positively charged AUNC-L^41^. Being ultra-small in size, AUNCs are like small drug molecules which enabling their homogeneous controlled interaction with Amp which in turn leads to a consistent release of both the agents. Interaction of Amp with AUNC-L resulted in a shift (peak at around ≈ 343 nm) in UV spectra which confirms the successful conjugation (Fig. 1a). Further, shift in the fluorescent emission spectrum of AUNC-L-Amp clearly indicates the conjugation of Amp on the surface of AUNC-L (Fig. 1d), which is also supported by previous reports^29^. Due to higher surface area to volume ratio the fabricated nanoclusters allows higher Amp loading efficiency on its surface (72%). FT-IR spectra depict the conformational changes in the lysozyme secondary structures after the formation of AUNC-L and AUNC-L-Amp. The IR spectra of AUNC-L-Amp reveal prominent peak shift in beta-turn, alpha helix, random coil, inter-molecular strand and side chain vibration which indicate successful conjugation of Amp on AUNC-L surface (Fig. 1b). The synthesis and conjugation were also confirmed from the results of DLS, TEM (Fig. 2), XRD (Fig. 1c), SERS (Fig. 1e) and zeta potential analysis. The AUNC-L-Amp demonstrates excellent biocompatibility towards human RBC and mouse fibroblast cells (L929) (Fig. 3 and Fig. S2). Interactions of mammalian cells with biomaterial are mostly mediated by the electrostatic interaction between positively charged biomaterials and negatively charged cell membrane^42^. In this study, the interaction between positively charged AUNC-L-Amp and mammalian cells does not result in substantial membrane disintegration. Surface chemistry, shape, size, surface activity, chemical composition, and solubility of nanomaterials play a crucial role in its biocompatibility and endocytosis-exocytosis patterns^42–45^. Lysozyme functionalization on gold nanoclusters introduce biocompatible functionalities with formation of more stable protein corona in biological fluids. Nanoclusters undergoes pinocytosis which are less cytotoxic than nanoparticles, as the bigger particles get wrapped in the process of endocytosis and causes increased cellular uptake and subsequent toxicity^46^. In this context, previous studies suggested that gold nanoparticles of less than 3 nm are found to be non-toxic in *in vivo* systems with a faster rate of exocytosis^47^. Unlike widely evaluated antibacterial silver nanomaterials, AUNCs does not involves in reactive oxygen species (ROS) mediated cell death^9^. ROS independent mode of action of AUNCs partly explains the minimum cytotoxicity of AUNC-L and AUNC-L-Amp. Previous reports suggested that lysozyme capped gold nanoclusters possess antibacterial activity against pan-drug-resistant *Acinetobacter baumannii* and vancomycin-resistant *Enterococcus*; but its activity against MRSA is not known^48^.

Functionalization of Amp on AUNC-L restores its efficacy against MRSA clinical isolates and increases the therapeutic index against nonresistant bacterial pathogens. Nanoclusters possess superior antibacterial action due to porin protein channels mediated pronounced permeation into the cell^19^. Owing to have thicker cell walls, gram + Ve bacteria are less susceptible to bactericidal nanomaterials. In this study, lysozyme coating on AUNC renders its activity even against gram + Ve bacteria. Lysozyme denature the peptidoglycan through hydrolyzes of β (1 → 4) linkages between N-acetylmuramic acid and N-acetyl-D-glucosamine residues. Unlike Gram − Ve cell wall, higher amount of peptidoglycan and absence of outer membrane in Gram + Ve cell wall, render them more susceptible towards lysozyme mediated hydrolysis. But, lysozyme resistant phenomena is common in Gram + Ve *Staphylococcus* species and O-acetyltransferase OatA gene was found as the major determinant for the same^49^. In this study, AUNC-L exhibited considerable antibacterial activity against nonresistant gram + Ve bacterial pathogens. On the other hand, both AUNC-L and Free-Amp fails to exhibit antibacterial activity against MRSA. Amp activity has been restored towards MRSA clinical isolates upon conjugation with AUNC-L (Tables S2–S7) & (Fig. 4). Amp functionalization on AUNC-L increases the concentration of Amp at the site of antibiotic action which in turn facilitates multivalent binding of Amp on bacterial cell wall and blocks the efflux pump^11^. Lysozyme directed multivalent binding, and Amp mediated subsequent cell wall disruptions resulted in profound permeation of AUNC-L-Amp into the bacterial cell. Amp irreversibly inhibits transpeptidase and prevents third and final stage of bacterial cell wall synthesis and binary fission^50^. Though gold is generally regarded as biologically inert, ions released from AUNCs inside the bacterial cell has been reported to inhibit ATPase activities. This in turn destabilizes membrane potential, permeability, respiration, and binding affinity of ribosome subunit to t-RNA. Further it effects the functions of sulfur containing proteins (Fe-S protein) and phosphorous containing compounds (DNA)^51^. AUNC-L-Amp capable of over counters the β-lactamase due to increased ratio of Amp at the site of action. AUNC-L-Amp complex withstand MRSA resistance even after multiple exposures (15 times) of sub-inhibitory concentrations (Table S7). Antibacterial study results suggest that both lysozyme and Amp retains their activity which is in agreement with previous reports^52^. The AUNC-L-Amp is also capable of killing antibiotic tolerant MRSA persister population upon awakening through cis-2-decenoic acid (cis-DA) exposure. SYTOX Green membrane permeability and persister killing kinetics assay results demonstrated significant decrease in the MRSA persister cell population (Fig. 5). Multivalent presentation, lysozyme-mediated cell wall lysis, increased permeation of AUNC-L-Amp and efflux pump dysfunction disrupts the bacterial persister cells^53^.

AUNC-L-Amp eradicated MRSA infections from diabetic wound which accounting for pronounced and quicker wound healing (Fig. 7). The nanohybrid crosses the dermal epithelium easily and reduces the MRSA burden (Fig. S5), which attributes to decreased levels of inflammatory cytokines (Figs S6 and S7). Presence of lysosome in nanohybrid also helps in wound healing process through the cationic influence on the cell membrane of keratinocytes and developing acidic pH^54^. Further, AUNC-L-Amp also kills systemic MRSA and increased the life span of infected mice (Fig. 6). Ultra-small size of AUNC-L-Amp allows a slower and sustained release of Au species unlike the burst release behavior of Au ions from bigger nanoparticles. This phenomenon protects the mammalian cells from unwanted damages through rapid excretion of nanoclusters from the body^9^.

In this study, we have developed a coherent broad spectrum antibacterial hybrid through Amp functionalization on lysozyme capped AUNCs. This system reverts MRSA resistance and significantly increased the anti-bacterial efficacy against non-resistant bacterial pathogenic strains. A significant decrease in the MRSA load on kidney and spleen was achieved through AUNC-L-Amp intraperitoneal administration, which results in improved survival rate of infected animals (Fig. 6). Topical application of AUNC-L-Amp also eradicates MRSA infection on diabetic wounds and resulted in acceleration of the healing process (Fig. 8). We conjecture that, MRSA resistance reversion and enhanced antibacterial activity of AUNC-L-Amp is due to; (i) increase in the Amp concentration at the site of action (ii) multivalent presentation and increased permeation of Amp (iii) lysozyme-mediated cell wall lysis (iv) bacterial efflux pump dysfunction (v) gold ion mediated destabilization (Fig. S8).

## Experimental section

### Bacterial strains and mammalian cells

*Escherichia coli* (MTCC 40), *Staphylococcus aureus* (MTCC 3160), *Staphylococcus epidermidis* (MTCC 425), *Bacillus subtilis* (MTCC 441), *Bacillus cereus* (ATCC 10702), *Micrococcus luteus* (MTCC 1538), *Klebsiella pneumoniae* (MTCC 3384), *Pseudomonas aeruginosa* (MTCC 424), *Proteus vulgaris* (MTCC 426) *were* either procured from ATCC, Manassas, USA or IMTECH, Chandigarh, India. Ten numbers of methicillin resistant *S. aureus* strains (MRSA 01–10) were obtained from Hayat Hospital, Guwahati, Assam, India. Clinical isolates were collected in accordance with the guidelines of Indian Council of Medical Research (ICMR), Govt. of India and a patient concern was taken. All the experimental protocols were approved by Institutional human ethical committee, Institute of Advanced Study in Science and Technology (IASST), Guwahati and performed in accordance with ICMR guidelines. Identification of MRSA was performed according to the recommendations of the National Committee for Clinical Laboratory Standards. L929, mouse fibroblast cell line was procured from National Center for Cell Sciences, Pune, Maharashtra, India and maintained as per supplier guidelines.

### Synthesis of lysozyme capped AUNCs (AUNC-L) and Ampicillin loading on AUNC-L

A 15 mg/ml freshly prepared lysozyme solution was obtained by dissolving desired amount of lyophilized lysozyme powder in Milli-Q water. The lysozyme solution was stirred at the speed of 500 rpm and maintained at 4 °C. Further, an aqueous solution of HAuCl_4_ was slowly introduced in the protein solution drop by drop and maintained the final concentration as 2 mM. The *p*H of the solution was maintained at ≈6.5 throughout the experiment. The stirring is continued until the color of the solution was changed to pale red color, which indicates the formation of lysozyme capped gold nanoclusters (AUNC-L). The solution was centrifuged at 4500 rpm for 5 min to segregate the large or agglomerated entities and the supernatant was filtered through 0.22 µm membrane. The resulting solution containing AUNC-L was subjected to freeze drying and mixed with Amp in ultra-pure water at 1:2 ratio respectively. The reaction mixture was stirred for 24 h under dark conditions at 4 °C. Further, the solution was centrifuged and washed with ultrapure water to remove the free drug, followed by lyophilization. The contents of Amp in supernatant was measured through UV−vis spectrophotometry to evaluate the Amp loading efficiency (LE %) by following the formulae: LE (%) = [*m* (total Amp) − *m* (Amp in supernatant)]/*m* (total Amp) × 100%.

### Biocompatibility studies

#### Interaction with human blood cells

The method for experiments on human blood cells was approved by the Institutional Human Ethical Committee (IHEC), IASST, Guwahati, Assam, India. An informed consent was taken from the human volunteer prior to collection of blood samples and the experiments were conducted in accordance with ICMR guidelines. Interaction of AUNC-L-Amp with human blood cells was evaluated through percentage hemolysis assay. FE-SEM analysis was further performed to evaluate the changes in RBC morphology upon interaction^55^.

#### Interaction with mammalian cells

MTT and colony forming assays were performed for Free-Amp, AUNC-L, and AUNC-L-Amp against L929 mouse fibroblast cell line according to standardized protocols^55^.

### *In vitro* antibacterial activity evaluation

#### Zone of inhibition (ZOI), Minimum inhibitory concentration (MIC) and Minimum bactericidal concentration (MBC)

The ZOI experiment for Free Amp, AUNC-L and AUNC-L-Amp (25 µg/ml) was performed by agar disk diffusion method^55^. The enhancement in antibacterial activity of the nonresistant pathogenic bacteria in response to the treatment of AUNC-L-Amp was quantified by the following equation: (B − A)/A × 100, here A and B denotes to ZOI for Free-Amp and AUNC-L-Amp respectively.

Broth micro dilution method was employed to evaluate the MIC for Free-Amp, AUNC-L-Amp against the bacteria mentioned in ZOI section^7^. After 48 h of incubation, the wells with no bacterial growth on treatment with Free-Amp, AUNC-L-Amp were sub cultured on nutrient agar plates by 0.2 ml of inoculum to determine the MBC^56^. Vancomycin (25 µg/ml) was kept as negative control for these experiment.

#### *In vitro* biofilm assay

The role of AUNC-L-Amp on destabilization of biofilm was evaluated by following crystal violet microtiter plate assay^55^.

#### Killing kinetics assay

To evaluate the killing rate of AUNC-L-Amp and Free-Amp, killing kinetics assay was performed^52^. Overnight cultures of MRSA1 and MRSA2 were taken in an approximate concentration of 2 × 10^5^ CFU/ml, treated with 16 µg/ml Free-Amp or AUNC-L-Amp and incubated at 37 °C with shaking. The number of viable bacteria was enumerated by analyzing samples at 0, 2, 4, 6, 8 & 12 h post treatment.

#### MRSA persister membrane permeability assay

MRSA persister membrane permeability evaluation was performed by SYTOX Green assay^57^.

#### Morphological characterization of bacteria

MRSA1 suspension containing 5 × 10^7^ CFU/ml was incubated with AUNC-L & AUNC-L-Amp (16 µg/ml) for 12 h, centrifuged (4500 rpm) at 4 °C for 10 min and washed with sterile PBS. Bacteria with no treatment were considered as control (for FE-SEM only). After the treatment period, bacterial cells from all the treatment groups were fixed with 3% glutaraldehyde solution and preceded for FE-SEM and confocal microscopic analysis.

### Activity against MRSA persister population

#### Persister isolation

To isolate persister cells, overnight cultures of MRSA1 and MRSA2 (cultured in tryptic soy broth at 37 °C) were treated with 10 × MIC (20 μg/mL) gentamicin for 4 h. Activation of the SOS response in the presence of gentamycin was exploited to isolate MRSA persister cells from planktonic population^24^.

#### Persister killing efficacy assays

Killing efficacy assays were performed following standardized protocol^55^. Concisely, persister cells were exposed to Free-Amp, and AUNC-L-Amp at a concentration of 16 μg/mL along with the fatty acid signaling molecule cis-DA. MRSA1 and MRSA2 persister cells were pelleted, resuspended in 50 mL of saline, and aliquots of 7 mL were subjected to one of the following treatments: Free-Amp (or) AUNC-L-Amp in combination with cis-DA (310 nM) in saline. Cultures were incubated for 24 h at 37 °C with shaking (220 rpm). Persister cell viability was determined at definite time interval (0, 10, 20, 30, 40, 50, 60, 120, 180, 240 min) by both spectrophotometrically and CFU count method.

### *In vivo* experiments

#### Animals

To conduct the *in vivo* experiments swiss albino mice and wistar rats were obtained from Chakraborty enterprises, Kolkata and housed at animal house of IASST (Temperature 24 ± 1 °C and 45–55% relative humidity), Guwahati. All the animals were free access to water and standard pellet diet from Provimi animal nutrition Pvt. Ltd. India throughout the experimental period. The experimental protocols were approved by Institutional animal Ethical Committee (IAEC) of IASST, Guwahati (IASST/IAEC/2015–16/808) and performed in accordance to Committee for the Purpose of Control and Supervision of Experiments on Animals (CPCSEA) guidelines.

#### Efficacy of AUNC-L-Amp in a systemic MRSA murine infection model

MRSA1 culture was grown in nutrient broth for 12 h and diluted to an OD_600_ of 0.6 at 37 °C with subsequent shaking, centrifugation and washing in PBS. Intraperitoneal injection of MRSA1 of 3.9 × 10^6^ CFU was given to Swiss albino male mice (n = 8; age: 4 weeks; weight: 22 ± 2 g). After 1 h of infection induction, the animals were treated with intraperitoneal injection of different treatments as follows-

- **G-I:** Animals with infection + PBS.
- **G-II:** Animals with infection + 25 mg/kg of Free-Amp.
- **G-III:** Animals with infection + 25 mg/kg of AUNC-L.
- **G-IV:** Animals with infection + 25 mg/kg of AUNC-L-Amp.

Post 23 h of drug treatment, 4 animals from each group were sacrificed, and the kidneys and spleens were harvested in a sterile condition, homogenized in PBS (pH: 7.4) and plated on nutrient agar for CFU determination. Drug treatments were continued every 24 h for remaining 4 animals up to 7 days to determine the survival rate.

#### Efficacy of AUNC-L-Amp in rat diabetic wound infection model

Male rats of Wistar strain weighing 200–220 g were selected for this study. Diabetes was induced to overnight fasted rats through single intraperitoneal injection of streptozotocin (STZ) at 55 mg/kg (dissolved in 0.1 M citrate buffer of pH 4.5). Diabetes was confirmed through measuring fasting blood glucose (FBG) levels three days post STZ injection. Animals having FBG levels > 250 mg/dl were selected and included in the study. After 4 days of diabetes induction, animals were anesthetized by intraperitoneal injection of ketamine and xylazine cocktail (80 & 10 mg/kg respectively) and wounds (1.5 cm in diameter) were created on the dorsal side with a surgical scalpel. The wounds were inoculated with MRSA1 (200 µl of 2 × 10^9^ CFU/ml) culture and covered with sterile cotton gauge. After 3 days of incubation, wound scrapings were collected to measure the CFU load for confirming infection establishment and following drug treatment was administered from 4^th^ day and continued for 20 days.

Grouping and drug treatment: (n = 6)

- **G-I:** Diabetic wounds with no infection and treated with PBS topically.
- **G-II:** Diabetic wounds with MRSA infection and treated with PBS topically.
- **G-III:** Diabetic wounds with MRSA infection and treated with Free-Amp topically at16 µg/ml.
- **G-IV:** Diabetic wounds with MRSA infection and treated with AUNC-L-Amp topically at16 µg/ml.

At different time intervals (3^rd^, 7^th^, 14^th^ & 21^st^ day) wound scrapings were collected to measure the CFU load. Blood was collected through retro-orbital route and serum was separated to measure the interleukins (IL-1β & TNF-α) levels using ELISA kits obtained from R&D systems. At the end of the drug treatment period, skin from the wounded area was collected from animals in different groups (At 21^st^ day for G-I & G-IV; 13^th^ day for G-II & 17^th^ day for G-III) for histopathology examination. Slides were stained with hematoxylin and eosin to observe the skin pathology under the light microscope.

The detailed description of remaining experimental procedures has been given in supplementary information.

## Electronic supplementary material

Supplementary information
