# Supplementary material for: Functionalization of β-lactam antibiotic on lysozyme capped gold nanoclusters retrogress MRSA and its persisters following awakening
Source: Sci Rep. 2018 Apr 10;8:5778. doi: 10.1038/s41598-018-22736-5 (PMC5893536; doi:10.1038/s41598-018-22736-5)
Supplement: Supplementary file 1 — Supplementary information [file 41598_2018_22736_MOESM1_ESM.docx]

**Functionalization of β-lactam antibiotic on lysozyme capped gold nanoclusters retrogress MRSA and its persisters following awakening**

Sanjeeb Kalita^1^^*, Raghuram Kandimalla^1^^*, Ashim Chandra Bhowal^2^*, Jibon Kotoky^1^, Sarathi Kundu^2^^*

^1^Drug Discovery Lab, Institute of Advanced Study in Science and Technology, Paschim Boragaon, Assam, Guwahati-781035, India.

^2^Soft Nano Laboratory, Institute of Advanced Study in Science and Technology, Paschim Boragaon, Assam, Guwahati-781035, India.

* **All the authors have equal contribution**

^ Corresponding authors: [sarathi.kundu@gmail.com](mailto:sarathi.kundu@gmail.com), [raghuram.pharma@gmail.com](mailto:raghuram.pharma@gmail.com) [sanju.kalita@gmail.com](mailto:sanju.kalita@gmail.com)

**Supporting information:**

**Materials and Methods:**

**Chemicals:**

Lysozyme (catalog No. 62971) and Gold (III) chloride trihydrate (HAuCl_4_) (catalog No. 520918), ampicillin (catalog No. A9518-5G), tetra methyl silane, 3-(4,5-Dimethylthiazol-2-yl)-2,5-Diphenyltetrazolium Bromide (MTT) (catalog No. M5655-1G), cis-2-decenoic acid (cis-DA) (catalog No. 19699), and streptozotocin (catalog No. S0130-1G) was purchased from Sigma-Aldrich, USA. SYTOX Green (catalog No. S7020) was purchased from thermo-fisher scientific. Cell culture media was procured from Invitrogen, USA. Bacterial culture media was purchased from Himedia, India. All the other chemicals used in this study were of analytical grade and obtained from Merck, Germany and Sigma-Aldrich, USA. Ultrapure Milli-Q water was used throughout the experiments. For IR and XRD experiments, thin films of lysozyme capped gold nanoclusters (AUNC-L) before and after conjugation with ampicillin were prepared on both Si and glass substrates. For this particular experiment, all the substrates were treated with a mixture of ammonium hydroxide (NH_4_OH, Merck, 30%), hydrogen peroxide (H_2_O_2_, Merck, 30%) and Milli-Q water (H_2_O:NH_4_OH:H_2_O_2_ = 2:1:1, by volume) for 5-10 min at 100 °C followed by drying at normal room temperature (25 °C). After that thin films were prepared by drop casting method followed by drying at room temperature (25 °C).

**Instruments:**

UV-Vis spectra were taken by using Shimadzu UV-1800 UV-Vis spectrophotometer, and the photoluminescence emission spectra were recorded by using fluorescence spectrometer (Cary Eclipse). The attenuated total reflection fourier transform infrared (ATR-FTIR) spectroscopy data were taken using NICOLET 6700 (Thermo Fisher) spectrophotometer within the wave number range of 4000 cm^−1^ - 500 cm^−1^ at 4 cm^−1^ resolutions. X-ray diffraction (XRD) measurements were carried out using D8 Advanced, Bruker, AXS (XRD) setup. The diffractometer consists of a Cu source (sealed tube) followed by a Göbel mirror to select and enhance Cu Kα radiation (λ=1.54 Å). The scattered beam was detected using NaI scintillation (point) detector. SERS spectra were taken using laser micro Raman system and excited using the 633 nm excitation laser at 257 Mw (Horiba Jobin Vyon, Model Lab Ram HR). Ultra high-resolution transmission electron microscope (HR-TEM) (Zeol2100) was used to evaluate the morphology, size, and purity of the nanoclusters. Morphology of the red blood cells (RBC) and bacteria was observed under field emission scanning electron microscopy (FE-SEM) (Carl-Zeiss, SIGMAVP, Japan). Fluorescent images of bacteria were taken with confocal laser scanning microscope (Zeiss LSM800).

**Biocompatibility studies:**

**Interaction with human blood cells:**

***% Hemolysis assay:*** AUNC-L-Amp at various concentrations (0.01, 0.1 & 1 mg/ml) were incubated with 10 ml of human red blood (1:9 dilution with saline) for different time intervals (10 min, 1 h, 6 h, 12 h & 24 h) at 37 ºC. Blood (1:9 dilutions) with distilled water & saline separately used as positive and negative controls. After the incubation period, the blood reaction mixture was centrifuged at 1500 rpm for 10 min, and the supernatant was measured for free hemoglobin in a UV-Vis spectrophotometer at 540 nm. ^S1^ The percentage hemolysis induced by the test samples were calculated according to the following formula-

(Abs _sample_ - Abs _–ve control_)

% Hemolysis = X 100

(Abs _+ve control_ - Abs _–ve control_)

***FE-SEM analysis:*** To further evaluate the RBC morphology, respective solutions of the treatment groups after 24 h of incubation were processed for FE-SEM analysis. Briefly, the blood pellets were fixed in 3% glutaraldehyde solution for 4 h and washed with 0.2 M phosphate buffer saline (PBS) of pH 7.4. Further, the cells were incubated with 0.2 M PBS (pH 7.4) for 6 h at room temperature and continued towards dehydration with acetone (30, 50, 70, 90 & 100 %). With each concentration of acetone, the cells were incubated for 15 min at room temperature. In the next step, the cells were treated with dry acetone for 15 min and further incubated with tetramethyl silane for 15 min at 4 ºC. The cells were kept in a desiccator for overnight and proceed for FE-SEM analysis. ^S2^

**Interaction with mammalian cells:**

***MTT assay:*** L929, mouse fibroblast cell line was procured from National Center for Cell Sciences, Pune, Maharashtra, India and maintained as per supplier guidelines. 1 X 10^4^ cells/ well were cultured in 100 µl DMEM (Dulbecco’s Modified Eagle Medium) supplemented with 10% fetal bovine serum (FBS) and incubated for 24 h at 37 ºC in a CO_2_ incubator. The cultured cells were treated with different concentrations (5, 10, 20, 40, 50, 100, 150, 200 & 250 µg/ml) of Free-Amp, AUNC-L, and AUNC-L-Amp in FBS-free DMEM and incubated further for 24 h and the cell viability was assayed using MTT method. ^S3^

***Colony forming assay:*** Free Amp, AUNC-L, AUNC-L-Amp (250 µg/ml) treated L929 cells were trypsinised, counted for 1000 cells (Automated cell counter, Invitrogen, USA) and seeded in 25 cm^2^ cell culture flasks. Untreated cells, treated with serum-free culture media was kept as negative control. After 7 days of incubation, the score for viable colonies were evaluated. Colonies were fixed with 0.25 % methylene blue in 75% carbinol and the colonies having a minimum of 50 cells has been taken into consideration. Platting efficiency (PE) and surviving fraction were calculated as per the standard protocol. ^S3^

The number of colonies that arise after treatment of cells, expressed in terms of platting efficiency (PE), is known as survival fraction (SF):

SF= No of colonies formed after treatment/ No of cells seeded X PE

No of colonies formed

Where PE (%) = X 100

No of cell seeded

**Persister cell state isolation and confirmation:**

To isolate persister cells, overnight cultures of MRSA1 and MRSA2 (cultured in tryptic soy broth at 37 °C) were treated with 10 × MIC (20 μg/mL) gentamicin for 4 h. Activation of the SOS response in the presence of gentamycin was exploited to isolate MRSA persister cells from planktonic populations. The gentamicin-tolerant cells were further treated for additional 4 h with 10 X MIC of gentamicin, vancomycin and ciprofloxacin to determine the tolerance of MRSA towards other antibiotics.

**Antibacterial assay**

**Evaluation of bacterial resistance against AUNC-L-Amp MIC value:**

To determine the resistance acquiring ability of bacterial pathogens (*S. aureus*, MRSA-I, and MRSA-II) towards AUNC-L-Amp this assay was conducted. Free-Amp was taken as reference in case of *S. aureus* where, MRSA-I and MRSA-II tested against AUNC-L-Amp alone. Briefly- the bacterial suspensions (1 X 10^7^ CFU/ml) were incubated with sub-lethal dose of AUNC-L-Amp and Free-Amp up to 15 passages. At different time intervals of exposure (1^st^, 5^th^, 10^th^ & 15^th^ day) the bacteria (1 X 10^7^ CFU/ml) were tested for MIC and compared with initial MIC values. ^S4^

***In vitro* biofilm inhibition assay:**

The role of AUNC-L-Amp on destabilization of biofilm was evaluated by following microtiter plate assay. ^S3^ 200 µl of nutrient broth with 10^7^ colony forming units (CFU)/ml^-1^ of the MRSA1 & MRSA2 along with 16 µg/ml AUNC-L-Amp were inoculated in sterile polystyrene microtiter plate wells and incubated in static mode for 48 h at 37 ºC. Wells with Free-Amp treatment were compared with AUNC-L-Amp treatment. Whereas, wells without any test substances treatment considered as positive control. After 48 h of incubation, the loosely attached bacteria were washed with sterile phosphate buffer saline (PBS) of pH 7.4. Further, wells were treated with 200 µl of 0.1 % crystal violet and incubated for 25 min followed by PBS washing and air drying. The biofilm-bound stain was dissolved in 200 µl of 95% ethanol, and absorbance was recorded at 590 nm.

**MRSA membrane permeability assay:**

MRSA1 and MRSA2 were grown to stationary phase and diluted with PBS to OD_600_ = 0.4 (~2 x 10^8^ CFU/mL). To 10 mL of the diluted MRSA1 and MRSA2 suspension, SYTOX Green was added to a final concentration of 5 μM and further incubated at dark condition for 30 min at room temperature. 50 μL of the MRSA/SYTOX Green mixture was added to each well of 96-well plates (Black, clear-bottom 96-well plates, Corning no. 3904) containing equal concentration of Free-Amp, AUNC-L and AUNC-L-Amp. The fluorescence was measured at room temperature for up to 4 h using a fluorescence microplate reader with emission and excitation wavelengths of 525 and 485 nm, respectively. All experiments were conducted in triplicate.^S5^

**Morphological characterization of bacteria:**

MRSA1 suspension containing 5 X 10^7^ CFU/ml was incubated with AUNC-L & AUNC-L-Amp (16 µg/ml) for 12 h, centrifuged (4500 rpm) at 4 ºC for 10 min and washed with sterile PBS. Bacteria with no treatment are considered as control (for FE-SEM only). After the treatment period, bacterial cells from all the treatment groups were fixed with 3% glutaraldehyde solution and proceeded for FE-SEM and confocal microscopic analysis.

**Reference for supporting information:**

(S1) Choudhury, A.J., Gogoi, D., Chutia, J., Kandimalla, R., Kalita, S., Kotoky, J., Controlled antibiotic-releasing *Antheraea assama* silk fibroin suture for infection prevention and fast wound healing. Surgery **159**: 539-47 (2016).

(S2) Kandimalla, R., Kalita, S., Choudhury, B., Devi, D., Kalita, D., Kalita, K., Dash, S., Kotoky, J. Fiber from ramie plant (*Boehmeria nivea*): A novel suture biomaterial. Mater Sci Eng C. **62**, 816-22 (2016).

(S3) Kalita, S., Kandimalla, R., Sharma, K.K., Kataki, A.C., Deka, D., Kotoky, J. Amoxicillin functionalized gold nanoparticles reverts MRSA resistance. Mater Sci Eng C*.* **61**, 720-27(2016).

(S4) Ikai, H., Odashima, Y., Kanno, T., Nakamura, K., Shirato, M., Sasaki, K. et al. Niwano Y. *In vitro* evaluation of the risk of inducing bacterial resistance to disinfection treatment with photolysis of hydrogen peroxide. Plos One **8,** (2013).

(S5) Wooseong, K., Annie, L.C., Rajmohan, R., Beth B.F., Frederick, M. A., Eleftherios, M. Identification of an Antimicrobial Agent Effective against Methicillin-Resistant *Staphylococcus aureus* Persisters Using a Fluorescence-Based Screening Strategy. PLoS One; **10**, e0127640 (2015).

**Supporting data:**


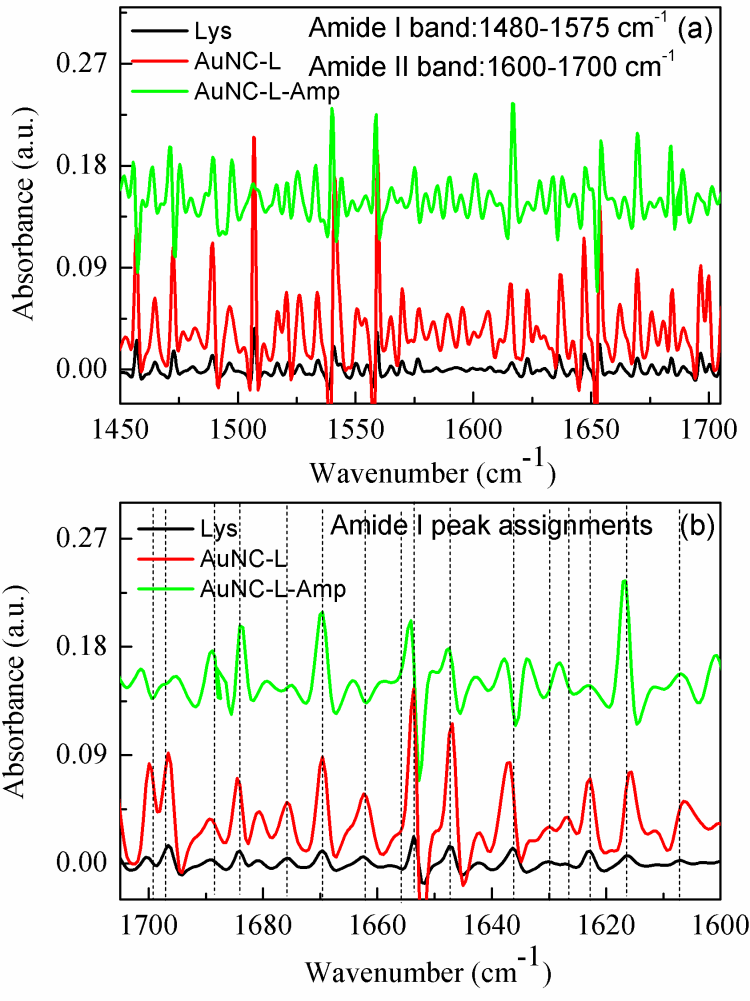


**Fig. S1.** **(a)** Second derivative IR spectra of Lys, AuNC-L and ANC-L-Amp showing the amide-I (1600-1700 cm^-1^) and amide-II (1480-1575 cm^-1^) bands. **(b)** Assignment of amide-I and II bands of Lys, AuNC-L and AuNC-l-Amp respectively. (Dotted lines indicate different peak positions corresponding to the lysozyme secondary structures).

**
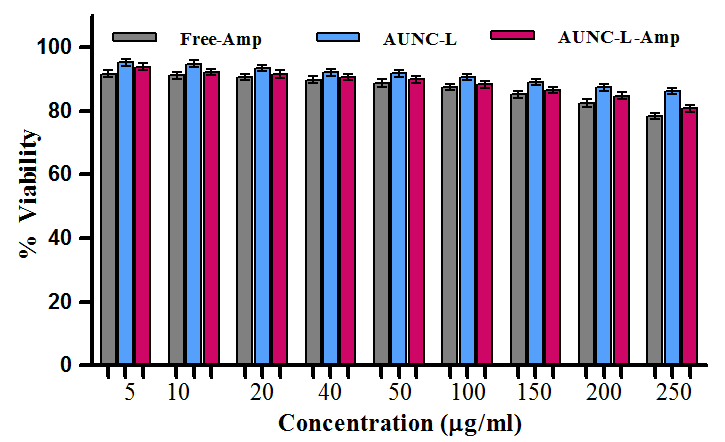
**

**Fig. S2.** MTT assay results showing % cell viability of L929 cells post 24 h incubation with different treatments at various concentrations (5- 250 µg/ml).

**
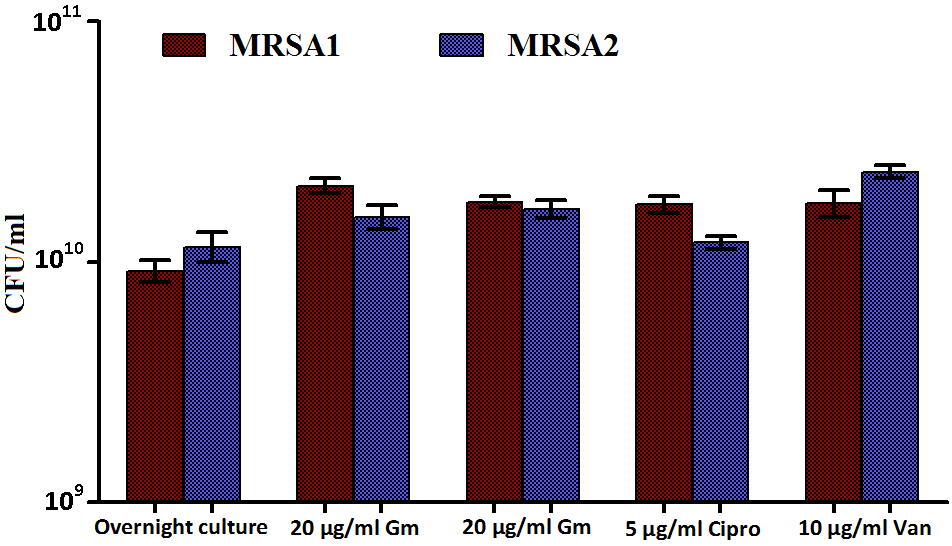
**

**Fig. S3.** Isolation of MRSA persisters. MRSA1 and MRSA2 overnight cultures were treated with 10 X MIC (20 μg/mL) gentamicin for 4 h and the titer of viable cells was determined. After the 4 h treatment with gentamicin, the culture was treated with additional antibiotics at the indicated concentrations (10 X MIC) for an additional 4 h, followed by once again determining the titer of viable cells. Results are shown as means ± s.d.; n = 3. Gm: gentamicin, Van: vancomycin, Cipro: ciprofloxacin.

**
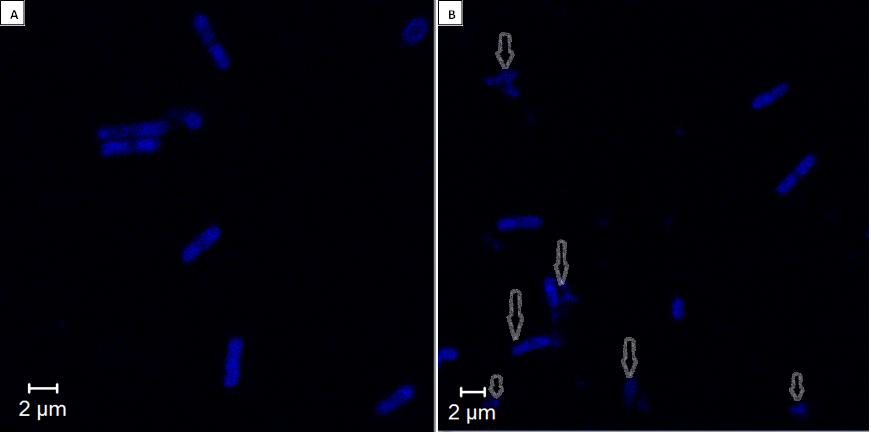
**

**Fig. S4.** Confocal images of MRSA treated with AUNC-L (A) and AUNC-L-Amp (B). Arrow marks: Dead bacteria.


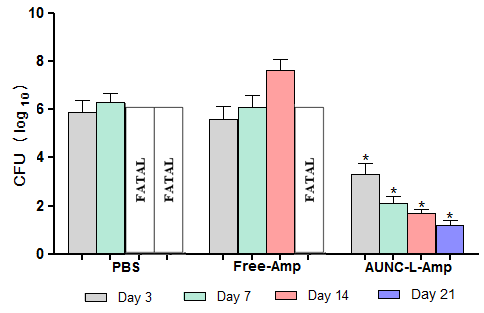


**Fig. S5.** MRSA load by means of CFU from wounded area at different time intervals post drug treatment. All the results were expressed in mean ± S.D. * p ≤ 0.05 in comparison with PBS treated group. ^ p ≤ 0.05 in comparison with Free-Amp treated group.


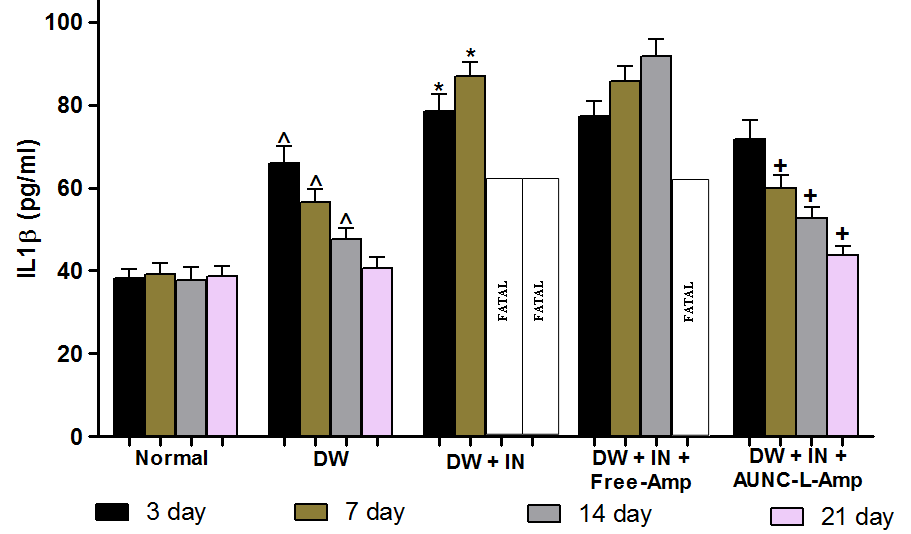


**Fig. S6.** Serum levels of Il-1β at different time intervals post drug treatment. All the results were expressed in mean ± S.D. ^ p ≤ 0.05 in comparison with the normal group. * p ≤ 0.05 in comparison with the DW group. **+** p ≤ 0.05 in comparison with DW + IN group. Abbreviations: DW: Diabetic wound; IN: MRSA infection.


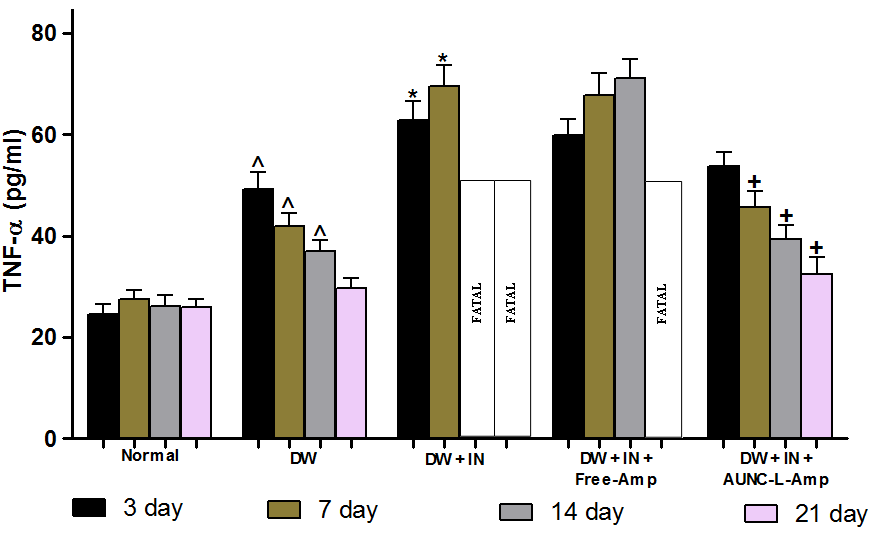


**Fig. S7.** Serum levels of TNF-α at different time intervals post drug treatment. All the results were expressed in mean ± S.D. ^ p ≤0.05 in comparison with the normal group. * p ≤ 0.05 in comparison with the DW group. **+** p ≤ 0.05 in comparison with DW + IN group. Abbreviations: DW: Diabetic wound; IN: MRSA infection.

**
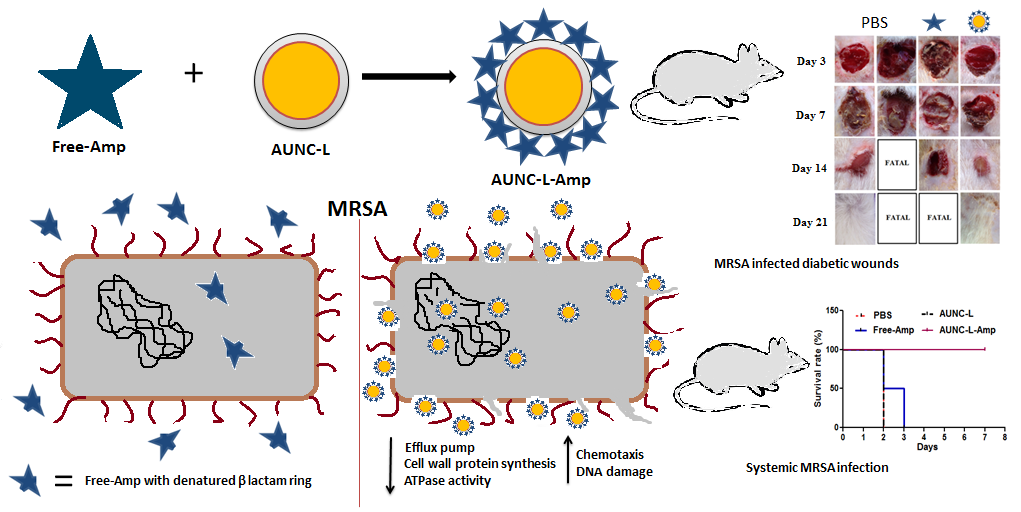
**

**Fig. S8.** Schematic representation of proposed mechanism of action of AUNC-L-Amp against MRSA.

**Table S1.** Assignment of FTIR peaks for Lys, AUNC-L and AUNC-L-Amp

| **S. No** | **Band Assignment** | **Lys ( cm^-1^)** | **AUNC-L  ( cm^-1^)** | **AUNC-L-Amp  (cm^-1^)** |
| --- | --- | --- | --- | --- |
| 1 | Anti-parallel beta sheet | 1700 | 1699 | 1699 (very weak) |
| 2 | Anti-parallel beta sheet | 1696 | 1695 | 1995 |
| 3 | Beta turn | 1689 | 1688 | 1688 |
| 4 | Beta turn | 1684 | 1684 | 1684 |
| 5 | Beta turn | 1676 | 1676 | 1675 |
| 6 | Beta turn | 1669 | 1669 | 1669 |
| 7 | 3_10_ Helix | 1662 | 1662 | 1664 |
| 8 | Alpha helix | 1657 | 1655 | 1655 |
| 9 | Random coil | 1653 | 1651 | 1651 |
| 10 | Random coil | 1647 | 1646 | 1643 |
| 11 | Beta sheet | 1636 | Absent | Absent |
| 12 | Beta sheet | 1630 | Absent | Absent |
| 13 | Intra-molecular beta strand | 1627 | 1627 | 1628 |
| 14 | Beta sheet | 1622 | 1627 | 1622 |
| 15 | Intermolecular beta strand | 1616 | Absent | 1617 |
| 16 | Side chain vibration | 1606 | 1607 | 1607 |

**Table S2.** *In vitro* zone of inhibition of Free-Amp, AUNC-L, and AUNC-L-Amp against non-resistant bacterial strains.

| **S.no** | **Bacterial Strains** |  | **Zone of inhibition (mm)** | | | |
| --- | --- | --- | --- | --- | --- | --- |
|  |  | **AUNC-L** | | **Free-Amp** | **AUNC-L-Amp** | **Increase in fold area %** |
| 1 | *E. coli* | 03 ± 0.17 | | 18 ± 0.47 | 34 ± 0.54 | 89 |
| 2 | *S. aureus* | 03 ± 0.14 | | 23 ± 0.39 | 37 ± 0.67 | 61 |
| 3 | *S. epidermidis* | 02 ± 0.11 | | 22 ± 0.43 | 35 ± 0.72 | 59 |
| 4 | *B. subtilis* | 05 ± 0.13 | | 24 ± 0.37 | 36 ± 0.66 | 50 |
| 5 | *B. cereus* | 06 ± 0.15 | | 23 ± 0.38 | 41 ± 0.79 | 78 |
| 6 | *M. luteus* | 05 ± 0.12 | | 16 ± 0.42 | 27 ± 0.61 | 69 |
| 7 | *K. pneumoniae* | 02 ± 0.06 | | 12 ± 0.39 | 19 ± 0.53 | 58 |
| 8 | *P. aeruginosa* | 02 ± 0.07 | | 16 ± 0.34 | 26 ± 0.58 | 63 |
| 9 | *P. vulgaris* | 02 ± 0.09 | | 17 ± 0.41 | 28 ± 0.64 | 65 |

All the results were expressed in mean ± S.D (n=3).

**Table S3.** *In vitro* zone of inhibition of Free-Amp, AUNC-L, and AUNC-L-Amp against MRSA clinical isolates.

| **S.no** | **Bacterial Strains** |  | **Zone of inhibition (mm)** | | | |  |
| --- | --- | --- | --- | --- | --- | --- | --- |
|  |  | **AUNC-L** | | **Free-Amp** | **AUNC-L-Amp** | **Increase in fold area %** | **Vancomycin** |
| 1 | MRSA1 | 02 ± 0.04 | | 03 ± 0.21 | 23 ± 0.52 | 666 | 36 ± 0.51 |
| 2 | MRSA2 | 02 ± 0.03 | | 03 ± 0.34 | 21 ± 0.46 | 600 | 42 ± 0.35 |
| 3 | MRSA3 | 03 ± 0.04 | | 04 ± 0.27 | 15 ± 0.48 | 275 | 47 ± 0.37 |
| 4 | MRSA4 | 02 ± 0.02 | | 05 ± 0.38 | 13 ± 0.56 | 160 | 33 ± 0.06 |
| 5 | MRSA5 | 01 ± 0.03 | | 05 ± 0.25 | 16 ± 0.45 | 220 | 34 ± 0.43 |
| 6 | MRSA6 | 02 ± 0.02 | | 03 ± 0.32 | 18 ± 0.51 | 500 | 47 ± 0.56 |
| 7 | MRSA7 | 02 ± 0.03 | | 02 ± 0.28 | 11 ± 0.48 | 450 | 41 ± 0.37 |
| 8 | MRSA8 | 03 ± 0.02 | | 04 ± 0.41 | 15 ± 0.58 | 275 | 38 ± 0.24 |
| 9 | MRSA9 | 03 ± 0.04 | | 03 ± 0.26 | 18 ± 0.54 | 500 | 51 ± 0.21 |
| 10 | MRSA10 | 02 ± 0.03 | | 05 ± 0.43 | 17 ± 0.47 | 240 | 33 ± 0.34 |

All the results were expressed in mean ± S.D (n=3).

**Table S4.** Minimum inhibitory concentration (MIC) and Minimum bactericidal concentration (MBC) of Free-Amp and AUNC-L-Amp against non-resistant bacterial strains.

| **S.no** | **Bacterial Strains** | **MIC/MBC (µg/ml)** | |  |
| --- | --- | --- | --- | --- |
|  |  | **Free-Amp** | **AUNC-L-Amp** | |
| 1 | *E. coli* | 6/8 | 1/2 | |
| 2 | *S. aureus* | 2/4 | 1/1 | |
| 3 | *S. epidermidis* | 2/4 | 1/1 | |
| 4 | *B. subtilis* | 32/64 | 8/16 | |
| 5 | *B. cereus* | 32/64 | 8/8 | |
| 6 | *M. luteus* | 64/128 | 16/32 | |
| 7 | *K. pneumoniae* | 128/256 | 64/128 | |
| 8 | *P. aeruginosa* | 64/128 | 32/64 | |
| 9 | *P. vulgaris* | 32/64 | 16/16 | |

All the treatments were performed in triplicate.

**Table S5.** Minimum inhibitory concentration (MIC) and Minimum bactericidal concentration (MBC) of Free-Amp and AUNC-L-Amp against MRSA clinical isolates.

| **S.no** | **Bacterial Strains** | **MIC/MBC (µg/ml)** | |  |  |
| --- | --- | --- | --- | --- | --- |
|  |  | **Free-Amp** | **AUNC-L-Amp** | **Vancomycin** | |
| 1 | MRSA1 | -- | 16/32 | 0.5 | |
| 2 | MRSA2 | -- | 16/32 | 0.5 | |
| 3 | MRSA3 | -- | 128/256 | 1.5 | |
| 4 | MRSA4 | -- | 128/128 | 1 | |
| 5 | MRSA5 | -- | 128/256 | 0.5 | |
| 6 | MRSA6 | -- | 32/64 | 1.5 | |
| 7 | MRSA7 | -- | 64/64 | 0.5 | |
| 8 | MRSA8 | -- | 128/128 | 1 | |
| 9 | MRSA9 | -- | 32/64 | 1.5 | |
| 10 | MRSA10 | -- | 64/128 | 2 | |

All the treatments were performed in triplicate.

**Table S6.** Biofilm inhibitory activity of Free-Amp and AUNC-L-Amp against four MRSA clinical isolates evaluated through crystal violate microtiter plate assay.

| **S. no** | **Bacterial strains** | **% Biofilm Inhibition** | |
| --- | --- | --- | --- |
|  |  | **AUNC-L-Amp (16 µg/ml)** | **Free AMP (16 µg/ml)** |
| 1 | MRSA1 | 95% | 9% |
| 2 | MRSA2 | 95% | 8% |
| 3 | MRSA3 | 92% | 11% |
| 4 | MRSA4 | 88% | 10% |

All the treatments were performed in triplicate.

**Table S7.** Minimum inhibitory concentrations (MICs) of bacteria on 1^st^, 5^th^, 10^th^ and 15^th^ day on exposure to anti-bacterial agent’s sub lethal dose.

| S.no | Treatment | MIC (µg/ml) | | | | |
| --- | --- | --- | --- | --- | --- | --- |
|  |  | Initial | 1^st^ day | 5^th^ day | 10^th^ day | 15^th^ day |
| *Staphylococcus aureus* | | | | | | |
| 1 | Free-Amp | 2 | 2 | 6 | 8 | 16 |
| 2 | AUNC-L-Amp | 1 | 1 | 1 | 1 | 1 |
| MRSA1 | | | | | | |
| 1 | AUNC-L-Amp | 16 | 16 | 16 | 16 | 16 |
| MRSA2 | | | | | | |
| 1 | AUNC-L-Amp | 16 | 16 | 16 | 16 | 16 |

All the treatments were performed in triplicate.
